# Development of a Mobile Game to Influence Behavior Determinants of HIV Service Uptake Among Key Populations in the Philippines: User-Centered Design Process

**DOI:** 10.2196/13695

**Published:** 2019-12-20

**Authors:** Charlotte Hemingway, Emmanuel S Baja, Godafreda V Dalmacion, Paul Mark B Medina, Ernest Genesis Guevara, Tyrone Reden Sy, Russell Dacombe, Claire Dormann, Miriam Taegtmeyer

**Affiliations:** 1 Department of International Public Health Liverpool School of Tropical Medicine Liverpool United Kingdom; 2 Institute of Clinical Epidemiology National Institutes of Health University of the Philippines Manila Philippines; 3 Department of Vector Biology Liverpool School of Tropical Medicine Liverpool United Kingdom; 4 Tropical Infectious Diseases Unit Royal Liverpool University Hospital Liverpool United Kingdom

**Keywords:** HIV, video games, health communication, persuasive communication, games, experimental, user-centered design

## Abstract

**Background:**

Opportunities in digital distribution place mobile games as a promising platform for games for health. However, designing a game that can compete in the saturated mobile games market and deliver persuasive health messages can feel like an insurmountable challenge. Although user-centered design is widely advocated, factors such as the user’s subject domain expertise, budget constraints, and poor data collection methods can restrict the benefits of user involvement.

**Objective:**

This study aimed to develop a playable and acceptable game for health, targeted at young key populations in the Philippines.

**Methods:**

Authors identified a range of user-centered design methods to be used in tandem from published literature. The resulting design process involved a phased approach, with 40 primary and secondary users engaged during the initial ideation and prototype testing stages. Selected methods included participatory design workshops, playtests, playability heuristics, and focus group discussions. Subject domain experts were allocated roles in the development team. Data were analyzed using a framework approach. Conceptual frameworks in health intervention acceptability and game design guided the analysis. In-game events were captured through the Unity Analytics service to monitor uptake and game use over a 12-month period.

**Results:**

Early user involvement revealed a strong desire for online multiplayer gameplay, yet most reported that access to this type of game was restricted because of technical and economic constraints. A role-playing game (RPG) with combat elements was identified as a very appealing gameplay style. Findings guided us to a game that could be played offline and that blended RPG elements, such as narrative and turn-based combat, with match-3 puzzles. Although the game received a positive response during playtests, gameplay was at times perceived as repetitive and predicted to only appeal to casual gamers. Knowledge transfer was predominantly achieved through interpretation of the game’s narrative, highlighting this as an important design element. Uptake of the game was positive; between December 1, 2017, and December 1, 2018, 3325 unique device installs were reported globally. Game metrics provided evidence of adoption by young key populations in the Philippines. Game uptake and use were substantially higher in regions where direct engagement with target users took place.

**Conclusions:**

User-centered design activities supported the identification of important contextual requirements. Multiple data collection methods enabled triangulation of findings to mediate the inherent biases of the different techniques. Game acceptance is dependent on the ability of the development team to implement design solutions that address the needs and desires of target users. If target users are expected to develop design solutions, they must have adequate expertise and a significant role within the development team. Facilitating meaningful partnerships between health professionals, the games industry, and end users will support the games for health industry as it matures.

## Introduction

### The Philippines’ HIV Epidemic

The Philippines has the fastest growing HIV epidemic in the Southeast Asia region. The dominant mode of transmission reported in the Philippines is sexual contact among males who have sex with males (MSM) and transgender women (TGW). As of 2017, the Joint United Nations Programme on HIV and AIDS reported the HIV prevalence rate at 0.1% for general adult population (aged 15-49 years), 0.3% among young men (aged 10-24 years), 4.9% among MSM, and 1.7% among TGW [1]. Specific data for TGW are limited [2]; however, a cross-sectional study in Cebu City in 2015 showed an 11.8% HIV prevalence rate in this group [3]. Cases are forecast to triple in the next 10 years, with the majority of new infections among young MSM (aged 15-24 years) [4]. Since the first reported case in 1984, the National HIV/AIDS and Antiretroviral Therapy Registry of the Philippines has confirmed 59,135 cases as of September 2018, of which 28% were aged between 15 and 24 years and 51% were aged between 25 and 34 years at the time of diagnosis. The proportion of HIV-positive cases in the 15- to 24-years age group has almost tripled in the last 10 years [5].

Young key populations worldwide pose a complex public health challenge; transmission rates are high, diagnosis is often delayed, and linkage to care and treatment is poor among those found to be infected [6]. Barriers to HIV services vary across contexts. In the Philippines, intrapersonal and social barriers exist alongside health system and economic barriers. Recurring themes associated with barriers to HIV services in the literature include low perceived risk of HIV infection; fear of losing access or status in important social spheres as a repercussion of accessing HIV services; lack of awareness or negative perceptions of treatment; belief that clinics do not provide confidential or private services; and restricted access to testing services because of time constraints, economic constraints, and legal constraints for those aged under 18 years [7-11].

### Why Play Mobile Games?

As the HIV epidemic worsens, social changes are occurring for young people in the Philippines. Increased online connectivity, a growing economy, and prevalent mobile device use has changed the way people spend their time and socialize. This change has generated new possibilities for the public health sector to deliver targeted health messages [12]. Utilizing technology already popular among adolescents and young adults may provide access to individuals who do not otherwise engage with traditional forms of HIV education and advocacy [13]. Mobile games, in particular, offer a promising platform to address the knowledge gaps, perceptions, social pressure, and self-stigma that deter young key populations from accessing health services [14]. A recent meta-analysis of 54 digital games for healthy lifestyle promotion found small but significant effects on behavior, determinants, and clinical outcomes, demonstrating the potential benefits [15].

Mobile games contain structural elements that effectively engage users [16], and it is through these elements that mobile games could influence behavior determinants. Players can model health-related behavior and witness positive and negative outcomes within a safe environment. Well-designed narratives, integrated with the gameplay, can foster identification with the characters, thereby increasing a player’s sense of personal risk or self-efficacy in overcoming barriers to HIV services [17]. Positive portrayals of characters living with HIV may help form beliefs that players can also remain or become their desired self after a positive diagnosis. Influence may also be found from the complex interplay between digital gaming and social behavior [18]. Even a single-player mobile game can trigger meaningful social interactions, from recommending a new game to offering advice on how to complete a challenge. For example, a player may recommend the game to a peer or family member who they believe could benefit from the health-related content. Narratives and characters within the game could also trigger meaningful conversations within social groups. Such use of the game could generate social pressure about health-related behavior.

### A Need for User-Centered Design in Games for Health

Rittel and Webber [19] defined the term *wicked problem* as a design problem that cannot be solved in a stepwise problem-solving manner. Game development is riddled with wicked problems. They are inherently interactive, and any interactive component must be tested with users. Even a simple game has an interconnected system as its backbone; any change to 1 component of the game will have ramifications to all connected components. Furthermore, game development is bound by countless budget, technical, user, and market constraints. As a solution to wicked problems, some game developers turned to user-centered design [20]. User-centered design is an iterative process; using a range of research techniques, feedback is obtained from users at different stages of product development to ensure their needs and preferences are considered in the design [21]. This design approach was established in the software industry to identify and rectify usability issues and expanded for use in game design to evaluate experiential aspects, supporting the development of games that are both functional and fun. User involvement in game design may have benefits with regard to the effectiveness of the game, although the evidence is mixed when reviewed in the context of games for healthy lifestyle promotion [22]. The core principle behind this theory is that, for a game to be effective, it must be acceptable and appropriate. This can only be achieved through consultation with target users, especially if the developers do not share the same characteristics as them. User involvement is also predicted to improve user adoption and enable developers to critically reflect on the value and consequences of a game when vulnerable groups are involved [23]. Thus, user-centered design methods must encompass interpretation and emotional response to health-related content in the game as well as evaluating usability.

**Figure 1:**
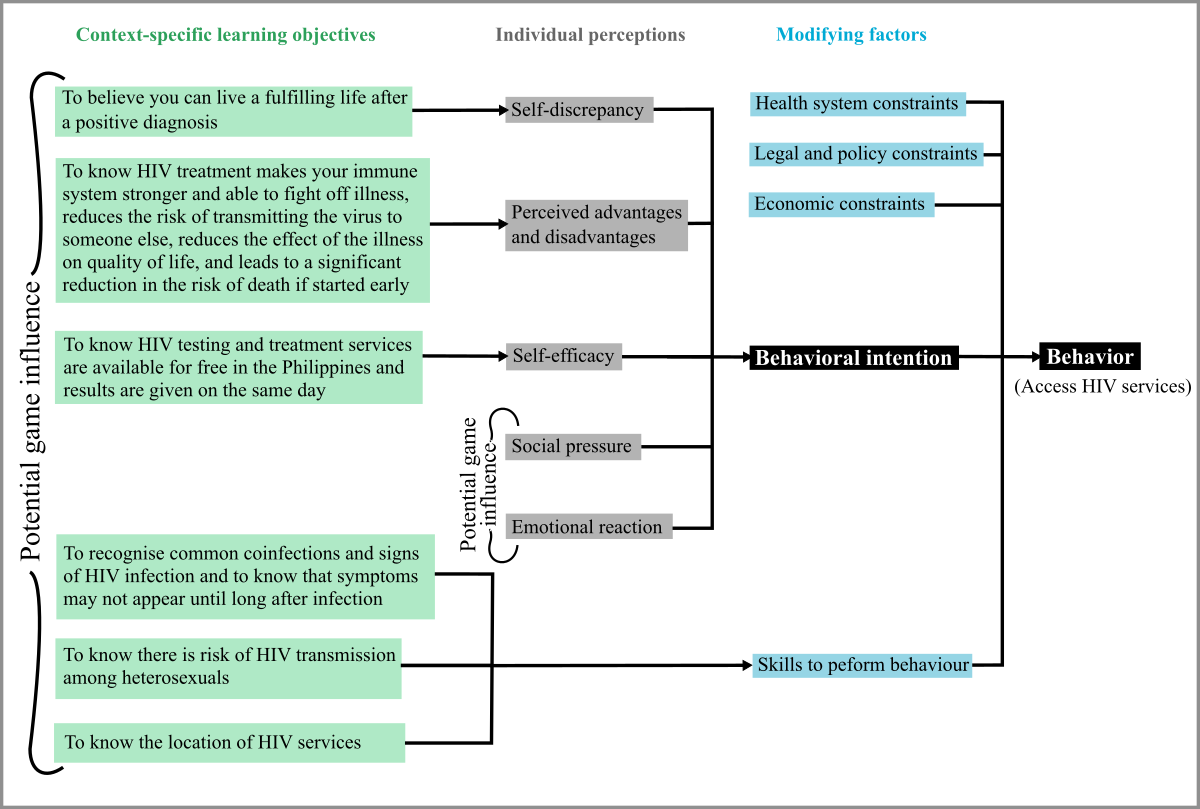
Integrative model of predictive behaviour and predicted game influence.

As the purpose of our game expanded beyond entertainment, we developed a theory of behavior change (Figure 1) to guide the design process [24]. Context-specific learning objectives were generated through a review of the literature and formative research with target users and HIV service providers in the regions of Davao and Manila. Data were collected through focus group discussions (FGDs) with HIV services providers, in-depth interviews with MSM and TGW, and an online survey on enablers and barriers to HIV services. Findings from the formative research are not included in detail in this paper. The framework references modifying factors relevant to the Philippines’ HIV testing context such as health system and legal and economic constraints. Given that the target audience for the game will be young MSM and TGW, we do not expect the game to impact modifying factors but note that these are important considerations in the evaluation of the game’s perceived effectiveness.

In this paper, we describe the user-centered design process for a mobile game titled *Battle in the Blood* and explore the effect of user-centered design techniques on the game’s acceptability as a health intervention and uptake among target users.

## Methods

### Overview

**Figure 2:**
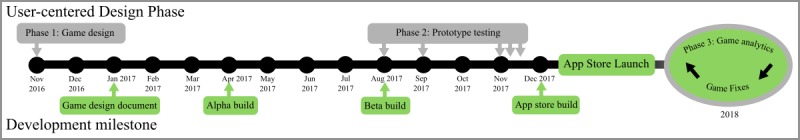
Development process.

*Battle in the Blood* was developed from 2016 to 2018 through a collaborative effort with experts from the United Kingdom and the Philippines. Final design choices were made by the game’s coproducers: a British behavioral scientist specializing in game design for health system benefit (CH); the director of an independent game development company, with over 10 years industry experience, based in Scotland; and a Filipino clinical epidemiologist (EB). The process was divided into 3 phases and aligned with development milestones for the game (Figure 2). Internal playtests were frequently conducted by members of the project team and their immediate networks. Change requests and bugs were recorded and shared with the development team using Google Docs.

### Selection of User Centered Design Methods

A literature review was conducted to identify a range of user-centered design methods to be used in a combined approach. Methods were selected based on their fit to the question the development team wanted to answer and the available resources. The resulting design process involved a phased approach, with primary and secondary users engaged during the initial ideation and prototype testing stages. Resources were prioritized to capture important contextual requirements and evaluate educational and experiential aspects of the game. Selected methods included participatory design workshops [25], extended playtests [20], playability heuristics [26], FGDs, and game analytics.

As the methods for each phase were distinct, recruitment, data collection, and analysis have been described for each. Data were collected in the cities of Manila and Davao. The study was part of a much larger project, which included assessment of a rapid diagnostic algorithm for HIV, that was being piloted in these cities.

### Phase 1: Game Design

To determine important contextual requirements and user preferences, design workshops with Filipino gamers were conducted during the initial specification and ideation stage. Subject experts in clinical practices for HIV and the Philippines HIV epidemic were allocated informant roles in the game development team.

#### Recruitment

Filipino individuals older than 18 years who regularly played digital games or were involved in game development or electronic sports, regardless of sexual identity, were invited to participatory design workshops via social networking sites, including Facebook and Steam forums. It was theorized that the game would need to be appealing and accessible to a range of gamer types and that gamer type would not be dictated by sexual identity. A total of 18 participants were divided into 2 groups (11 and 7). Participants were aged between 21 and 30 years and were a mixed group of MSM and non-MSM.

#### Data Collection

Group sessions were facilitated by UK and Filipino researchers (CH, EB, EG, and JD), in which (1) participants responded to questions by creating a human scatter graph indicating their level of agreement to different statements by their physical proximity to the statement placed on the floor, creating an instant visual of the group’s perception and experience of mobile games and games for health; (2) group discussions were held on design and technical enablers and barriers to digital gaming; and (3) participants were divided into teams (maximum 6 participants in a team) to develop pitches for games to promote HIV services, which they then presented and discussed. HIV clinic counselors were present at both workshops to answer any questions the participants had regarding HIV and provided information on HIV service provision in the Philippines. The session was audio recorded and transcribed, and photos were taken to record the human scatter graphs and visuals from the game pitches.

#### Analysis

Transcripts and session outputs were analyzed using a framework approach [27] to identify a set of recommended game features. A 4-day workshop was conducted with the full development team to translate the list of recommended game features into a game design document. Game features were also connected to the context-specific learning objectives outlined in Figure 1.

### Phase 2: Prototype Testing

Playtesting sessions and FGDs were conducted to assess the acceptability and playability of the beta game build.

#### Recruitment

Participants were older than 18 years and self-identified as MSM. This study aimed to ensure that the narratives and art style were appealing, relatable, and inoffensive to the target users. Participants were recruited using social networking sites, including Facebook, Grindr, Growlr, and PlanetRomeo, known as popular networking sites for the MSM and TGW community. Peer counselors from HIV testing services were also recruited as they were predicted to be an important user group for the game.

Overall, 5 FGDs and playtesting sessions involving a total of 22 participants were conducted between August 20, 2017, and November 18, 2017; 4 sessions were conducted in Manila (17 participants, all MSM) and 1 in Davao (5 participants, all peer counselors and MSM). Game changes and bug fixes were implemented between each testing session.

#### Data Collection

Sessions were facilitated by UK and Filipino researchers (CH, EB, EG, and JD). User testing was divided into 3 activities: (1) participants’ screens and faces were video recorded as they played the game for 30 to 45 min, (2) all animations in the game were played on a big screen and a short discussion was held by participants for each one, and (3) a group discussion was conducted on the perceived acceptability of the game.

#### Analysis

**Figure 3:**
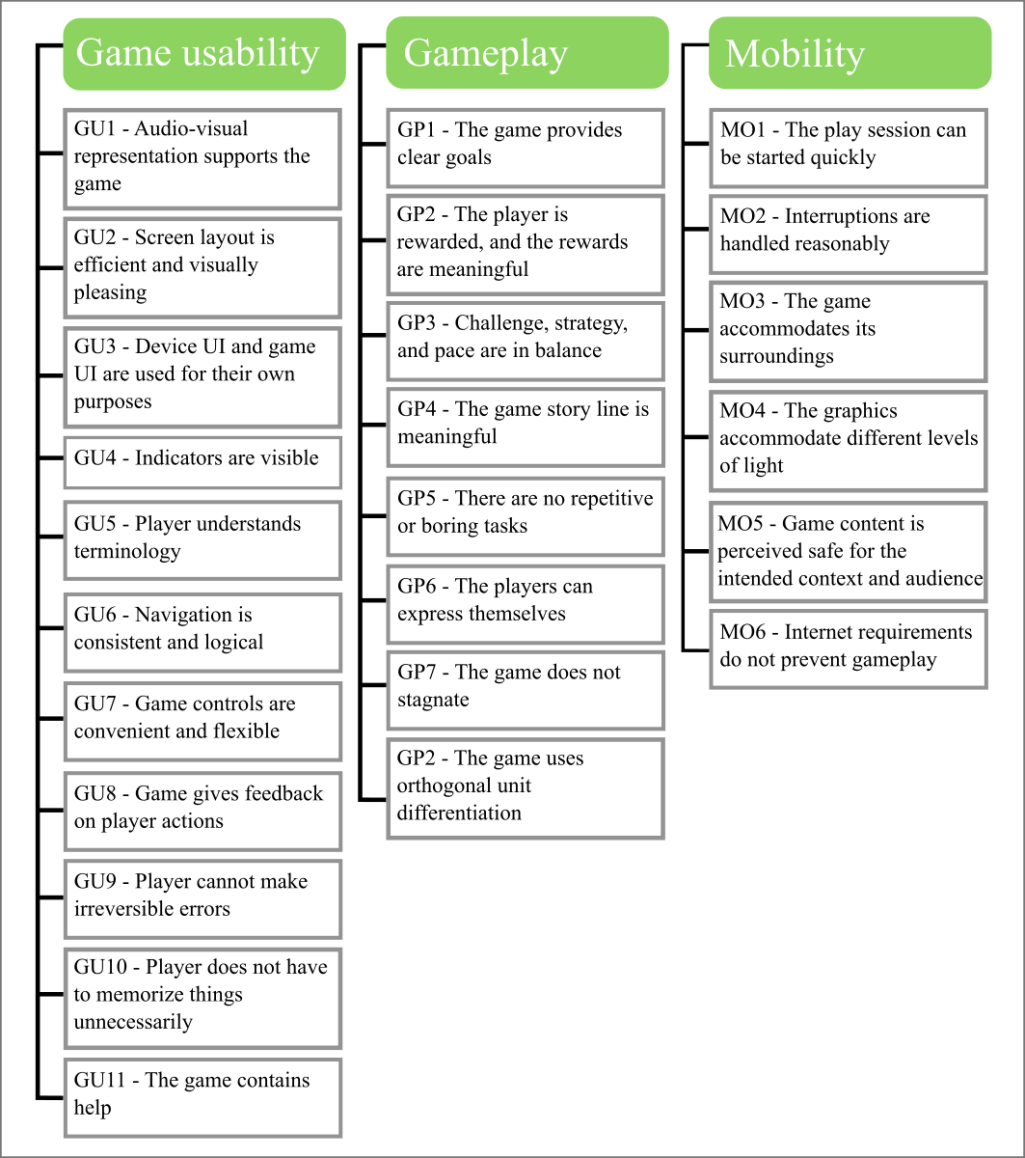
Playability heuristics for mobile game for health. UI: user interface.

Analysis of data from the game testing sessions was divided into 2 parts. The first part utilized playability heuristics to identify and fix design flaws. Playability heuristics are a set of qualities by which a game’s engagement and usability can be assessed. They are typically used by game developers and professional game testers. We adapted an existing list of playability heuristics for mobile games [26] to use as a coding framework to analyze the video recordings and transcripts (Figure 3). Screen recordings were primarily used to identify technical and usability issues, whereas transcripts were more conducive to recognizing different experiential aspects. For example, expressions of confusion by the player when they lost a level, especially when followed by repeated failed attempts, could be marked as a violation of gameplay heuristic GP1, “the game provides clear goals” (Figure 3). This is an example of what is termed a playability violation. Playability reports were developed independently by 2 researchers (CH and EG). The reports described each playability violation and gave recommendations for game improvements. The reports were compared and discussed by the game development team before agreeing on a final list of game changes. Where feasible, changes were implemented before the next game testing session. Software bugs and technical issues were also recorded, and fixes were implemented.

The second part of the data analysis utilized the transcription of the group discussions to assess the acceptability of the game as a health intervention. Data analysis was informed by a general inductive approach, aligning emerging themes identified in the transcripts with the following predetermined constructs of health intervention acceptability: affective attitude, burden, ethicality, intervention coherence, opportunity costs, perceived effectiveness, and self-efficacy [28].

#### Quality Assurance: Phases 1 and 2

All qualitative data collected were translated into English for analysis (EG), and the translation was checked for accuracy by members of the research team. Analysis was led by a UK researcher (CH) with regular consultation and input from the full project team to improve quality and depth. All design choices resulting from the data were reviewed by the development team before implementation.

### Phase 3: Game Analytics

#### Recruitment

Game use data were obtained from users who installed the game and gave permission for the app to access device storage. Geolocation data were obtained from users who granted access to their devices’ location data and played the game with GPS switched on. Information about why data were being gathered and reassurances that all data would be kept anonymous were presented on the app store page, and in the game when access was requested.

The game was made available on the Apple App Store and Google Play on November 27, 2017, and was officially launched during World AIDS Day celebrations in the Davao Region on December 1, 2017. Marketing events for the game included local television appearances by project staff, printed and online news articles, exhibitions at health- and game-related conferences in the National Capital Region and Davao Region, social media advertisements on Facebook and Twitter, and posters displayed in 4 HIV testing and counseling clinics (3 in Manila and 1 in Davao). The game analytics span a 12-month period from December 1, 2017, to December 1, 2018.

#### Data Collection

Data were automatically gathered from devices with the game installed through the Unity Analytics service, version 2017.1 by Unity Technologies. For data to be sent from the device, the user must have opened the game at least once while the device was connected to the internet. Events are stored locally on the device when the game is played offline and sent the next time the game is opened and the device is connected. All data points were stored in the Unity Data Store. The game metrics (Table 1) were exported and converted into a readable format for use in statistical analysis software.

**Table 1:** Game metrics.

Segment	Metrics
Active player metrics	Daily active users; monthly active users; and new users (unique device installs)
Session metrics	Sessions per day; sessions per user; total daily playing time; and total playing time per active user
Retention metrics	Day 1, 7, and 30 retention
Platform segment	Total Android and iOS users
Custom segments	Level events—reports each time a level is completed or failed, and the players end score; link event—reports the click-through rate of all in-game links to external information; question events—reports the response to all in-game questions, including a set of demographic questions; geolocation per session—reports the device’s current location at the start of a session to 2 decimal places; and app version

#### Analysis

Descriptive statistical analysis was conducted in Microsoft Excel, version 1812. Spatial analysis was conducted in QGIS, version 3.2.3.

### Ethical Assurance: All Phases

Legal advice was sought to ensure that management of game analytics complied with the 2018 European Union General Data Protection Regulation.

Ethical approval for the project was obtained from the ethics committees at the University of the Philippines College of Medicine and Liverpool School of Tropical Medicine (research protocol 16-017).

## Results

### Phase 1: Game Design

**Figure 4:**
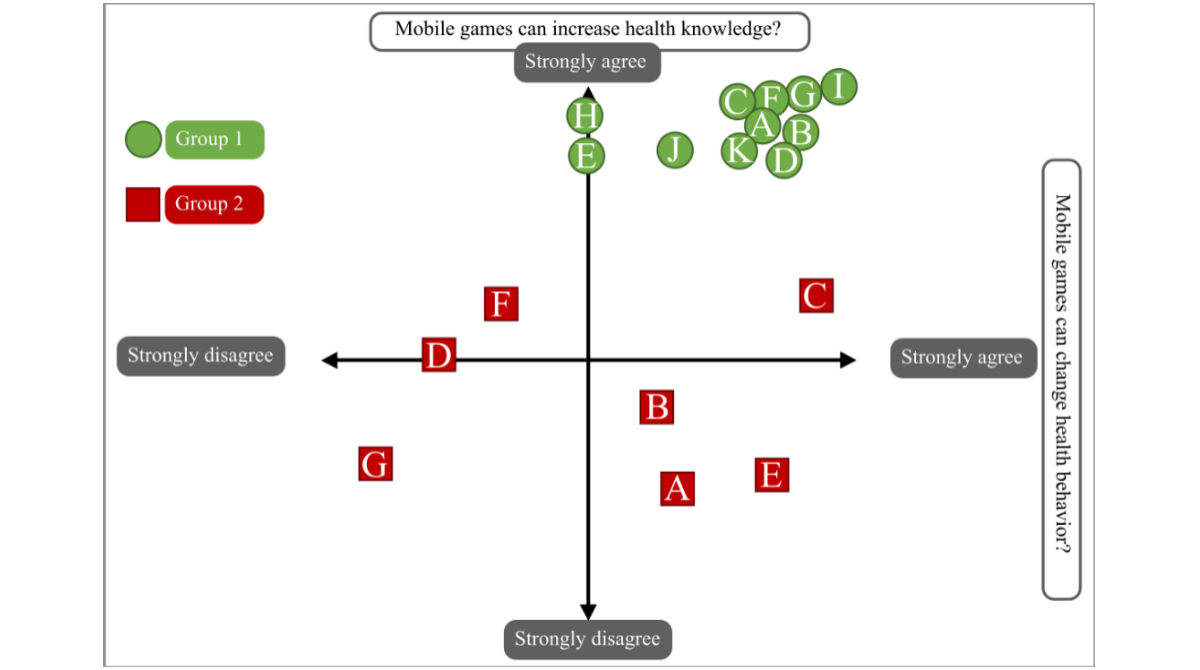
Human scatter graph on perceptions of the effectiveness of games for health behavior change.

Direction on game elements to support health-related behavior change first emerged through the human scatter graph activity. Figure 4 demonstrates that the 2 groups had varied perceptions on the effectiveness of games for health-related behavior change. Participants were questioned as to why they had chosen their place on the graph. Perceived limitations of the game’s effectiveness were centered around beliefs that changes in knowledge would not be sufficient to change behavior and that the use of technical language would make the game’s content inaccessible, as illustrated by the following quote:

I’m still not convinced with the knowledge part because, as with some health conditions, even with some health benefits and stuff, there will be some things that a player will not understand, especially if it’s scientific jargon...but I do believe that behavior can change especially if it’s immersive and it is an experience that will change your perspective on very different matters.

Group 2: Respondent B

Facilitators to behavior change centered around the use of immersive experiences that could change perspectives and generate social interactions between users, as illustrated by the following quote:

Because whenever I’m playing games, they would often ask me “Hi [name]! What’s that game? Is that available in iOS? Is it available in the Android Play Store?” Right after I told them about it, they would go check out the game and download it. So, it’s not just me but also them that learns from the game.

Group 1: Respondent J

During group discussion, the game’s narrative was identified as an important feature. If done well, it fostered an identification with the game characters and motivated the players to overcome challenges in the gameplay to witness the story unfold. The narrative was identified as the logical place to communicate why it was important to know one’s HIV status. Participants felt that the game should not provide technical information on HIV or HIV services but should focus on telling an emotionally driven story and provide the player choice over the narrative direction.

When the groups were divided into teams (2 teams in each group) to pitch their HIV advocacy games, 3 out of the 4 teams presented a similar concept of a hero sent on a quest to fight or evade the HIV virus, where story and role-playing game (RPG) elements intertwined and where reality and fantasy existed in the same space.

Multiplayer gameplay was reported to be a strong motivator for repeated gameplay and was perceived to play a role in the effectiveness of the game, as illustrated by the following quote:

...they say that no man is an island. So, I think it would affect the behaviour of the person if there is a community that pushes you. Someone playing alone will say “I’m just alone, nobody will care if I do this or do that.” But if you know somebody else is pushing you, it will affect your behaviour, it drives you as a person.

Group 1: Respondent I

However, participants also reported that access to these types of games was restricted because of the requirement of a stable internet connection:

I have external and internal factors why I leave the game. The external factor is whenever the game requires internet connection because the internet, the wireless data in the Philippines is not that good.

Group 1: Respondent E

This presented a dilemma for the game design. On the one hand, multiplayer gameplay was a very desirable game style among the participants as they could participate in discussions about HIV, behavior, and personal values and coconstruct their own narratives about a desired future. This was further evidenced by the market success of multiplayer mobile games in the Philippines. On the other hand, we did not have the resources to develop and maintain an online multiplayer platform, and known issues around internet connectivity would restrict access to the game.

#### Combining Feasibility With Desire

Findings from the phase 1 game design workshops directed the game’s overarching narrative and the integration of gameplay and storytelling. Players take on the role of the protagonist, entering the blood stream in an antiretroviral (ARV) pill capsule and battling anthropomorphic viruses, bacteria, and cancer cells using a weaponized mechanical suit. The gameplay combines match-3 puzzles with turn-based combat; players connect icons on a puzzle board that represent condoms, ARVs, healthy living, health care, and time. Connecting the icons builds up the player’s defense and attack status during the rounds of combat. The match-3 game style was selected because of the availability of prebuilt game assets and source code, which substantially reduced the development time and enabled the team to allocate time to the custom animations.

The gameplay is segmented with a series of 8 animated stories about people living with HIV that the player helps by progressing through the game. The difficulty of the levels and the types of enemy units are connected with the story line. For example, if the character in the story line is diagnosed with gonorrhea, an enemy unit representing gonococcus appears during the gameplay. In the game’s final mission, the player is introduced to a character with AIDS in critical condition; it is revealed that he has never been tested for HIV, treatment fails, and the character dies. Throughout the game, the player is awarded with fragments that combine to form an anting-anting, a traditional Filipino amulet believed to have magical powers. This amulet allows the player to travel back in time and change how the story ends for this character by encouraging him to undergo a HIV test.

The game can be played and completed offline. Online features include a global leader board, ranking players by their total score, and hyperlinks to websites containing information on HIV and HIV services in the Philippines. The file size of the game was also restricted to 53.4 MB on Android and 70 MB on iOS to reduce the risk of failed download attempts from the app stores. This in turn had ramifications for animations and sounds in the game, both of which can substantially increase the file size. To maintain a small file size, the number of sound effects and music tracks in the game was limited and animated stories were presented in a dynamic 2-dimensional comic book style, where static images moved across the screen to give the scenes depth and movement.

#### Integration of Context-Specific Learning Objectives

Table 2 details the design choices made in relation to the context-specific learning objectives outlined in Figure 1.

**Figure 5:**
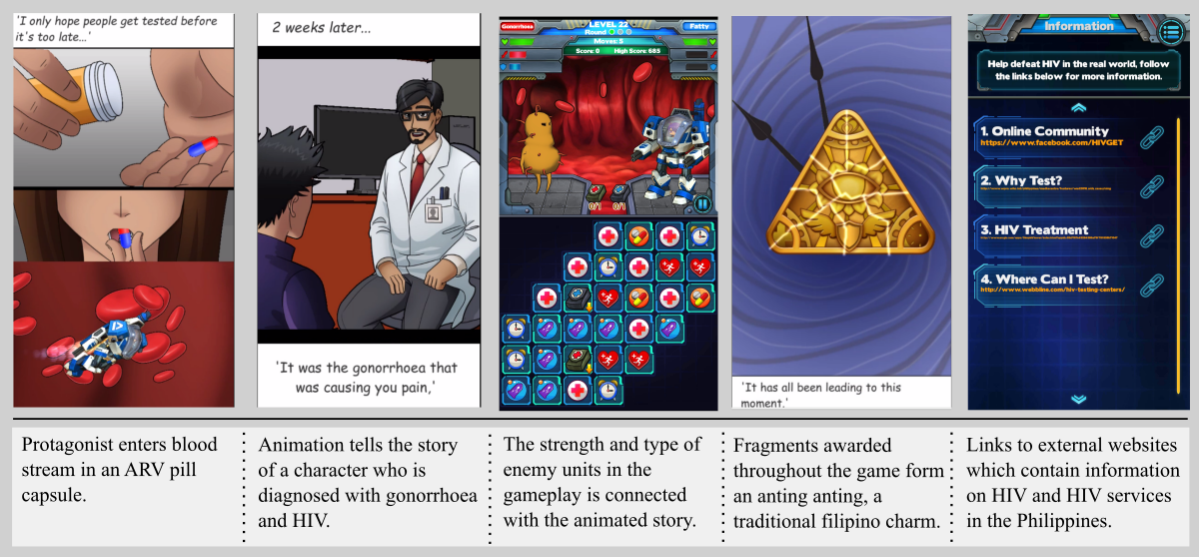
Screenshots Battle in the Blood v1.4.

Figure 5 uses screenshots from the game to illustrate some of the design choices made. A table summarizing the phase 1 design process (Multimedia Appendix 1) and the game design document, version 0.15, (Multimedia Appendix 2) are included as Multimedia Appendices.

**Table 2:** Theory of behavior change design choices.

Context-specific learning objective	Game design element
To believe that you can live a fulfilling life after a positive diagnosis	The animated stories depict people living with HIV pursuing their ambitions, being socially active, or spending time with their family. For example, a transgender character enters a beauty pageant and, as she takes her medication in the dressing room, the text reads, “I fought to be my true gender, I can win this fight too.” This particular story line was selected because of the cultural relevance pageantry has in the Philippines and the great esteem in which Filipinos hold the contestants.
To know that HIV treatment makes your immune system stronger and more able to fight off illness; reduces the risk of transmitting the virus to someone else; reduces the effect of the illness on quality of life; and, if started early, leads to a significant reduction in the risk of death	Antiretroviral tablets feature in every animated story except the one where the character dies. Treatment is also shown to improve the physical appearance of the characters in the later missions. Players can earn an extra life during gameplay by answering a multiple-choice question. Some questions address knowledge on treatment effects, and feedback containing the right answer is given. Hyperlinks to websites containing information on HIV treatment are included throughout the game, and the click-through rate is measured.
To know that HIV testing and treatment services are available for free in the Philippines and that results are given on the same day	In the animated stories, the words “Free HIV tests” appear in the background of all the clinic waiting room scenes. The rapid testing procedure is depicted in the first 3 animations. Multiple-choice questions address the availability of free testing and the time taken for the client to receive their results in the Philippines. Hyperlinks to websites containing information on HIV services in the Philippines are included throughout the game, and the click-through rate is measured.
To recognize common coinfections and signs of HIV infection and to know that symptoms may not appear until long after infection	Enemy units in the game include representations of gonorrhea, tuberculosis, hepatitis B, herpes, and cancer. Common signs of HIV infection are depicted in the animated stories, and some characters show no symptoms.
To know that there is a risk of HIV transmission among heterosexuals	The first animated story depicts a heterosexual couple. In several other animated stories, the character’s sexuality is ambiguous.
To know the location of HIV services	A hyperlink to a website containing the contact information and location of all testing sites in the Philippines is featured throughout the game.

### Phase 2: Prototype Testing

Table 3 summarizes the final agreed list of playability violations and the game changes made as a result.

#### Learning Achieved Predominantly Through Interpretation of the Animated Narratives

Participants repeatedly drew inferences between the presence of ARVs and the positive outcome in the short-animated stories, recognizing the unique benefits of treatment for each character, as illustrated by the following quote:

Ever since the guy was given the ARVs, his fear of him having STDs or socializing with people is now gone, and he was able to not be afraid to connect with other people.

GameTester_MSM

**Table 3:** Playability violations and game changes.

Playability heuristic	Evidence of violation	Game change
GP5: There are no repetitive or boring tasks	Participants felt bored and disengaged with the animation when the first half of the story line was repeated. Video footage demonstrated that players did not realize they could skip the recap by tapping anywhere on the screen.	On the second playthrough, a title screen appears with the text “Previously on *Battle in the Blood*…Skip?”
GU1: Audio-visual representations support the game	Players reported the icons as unintuitive because the color of the icons did not correspond with the combat status bars that they effected. Video footage showed players taking decisions based solely on the length of the chain, not the color of the icons, and becoming frustrated when they repeatedly lost a level.	The design of the icons was adjusted to correspond visually with the attack and defense bars.
GU11: The game contains help	Participants did not feel that the onboarding was comprehensive enough, and new mechanics in the game were not explained.	When a new game mechanic is triggered, a dialog box appears that explains the new mechanic using a small amount of text and images. This information can also be accessed via the game’s menu and via the level-pause screen.
GP6: The players can express themselves	Participants stated that they did not like the mechanical suit as they had spent time customizing the avatar but rarely saw it in the game as most of it was covered.	Avatar appears animated in the level-complete or -fail screen.
GP2: The player is rewarded, and the rewards are meaningful; GP5: There are no repetitive or boring tasks	Participants stated that they wanted to be able to earn in-game currency by completing the levels, which could be spent on upgrading or customizing their character. They also felt this would make the gameplay feel less repetitive.	Implementing an in-game currency system and custom character upgrades would have required additional resources and delayed the planned launch of the game. The change request was logged but not implemented for the pilot.

In addition to the inferred benefits of HIV treatment, participants also identified messages about the consequences of delayed access to HIV services. One participant reported that the game had changed his personal understanding of when to undergo a HIV test, as shown by the following conversation:

For me it’s better to test at the earliest, so you can know if you’re positive and prevent it earlier.

PhaseTwo_GameTester_MSM

So, before you played the game, you just know that you need to get tested when?

Interviewer

Only if you’re already having symptoms.

PhaseTwo_GameTester_MSM

Participants interpreted the stories based on their current knowledge and personal values. In 1 case, this led to the message of the story being repeatedly challenged:

...he was told that he has Gonorrhoea and he was then given pills to cure it and the HIV treatment. With that, he told himself that this could save his life and then could continue living his life normally. And then he was seen to be at the bar again. But it was somehow an off for me because it gave me the impression that just because there’s treatment, you can go on with your promiscuous activity all your life.

PhaseTwo_GameTester_MSM

Some counterarguments were expressed because of preexisting knowledge, namely, a lack of awareness that ARVs can reduce the viral load to the point where the individual is no longer infectious and social norms in which sexual promiscuity is perceived as an undesirable trait. Most participants concluded that the character in this particular animation was sexually promiscuous because the story centered around a nightclub, and he was diagnosed with HIV and gonorrhea. In this case, the narrative appeared to challenge stigmatizing perceptions around sexual promiscuity and its association with HIV.

Although participants felt that the health-related messages were reinforced in the turn-based combat levels, most of the learning outcomes were achieved through interpretation of the animated narratives and the discussions they triggered. A potential concern is that the animations were perceived to be disconnected from, and less engaging than, the gameplay. During the prototype testing session, all in-game animations were played in sequence through a projector, and participants intently watched and discussed each animation. Interpretation of the narrative may be significantly different by a user casually playing the game alone, with no direct incentive to focus on and analyze the content.

#### Identification With Game Characters

Animated narratives where the players shared a set of commonalities with the character diagnosed with HIV elicited a stronger emotional reaction than those where the characters did not share similar demographics or behaviors. The story of a young MSM who resorts to solicitation to afford to play games at an internet café was reported to be the most impactful story line by almost all game testers. This was likely because of the recruitment strategy, as most game testers were part of the young MSM community and had a strong interest in gaming:

Well it seems relatable for me because I’m a gamer. And I have a friend who does that thing sometimes. And thankfully he hasn’t got any HIV. It’s relatable to me because well, I know those who did, I experienced one but thankfully I haven’t got HIV.

GameTester_MSM

The game follows the stories of 8 different characters diagnosed with HIV. Each character is unique in its sexuality, gender, appearance, and behavior. Therefore, a user is likely to empathize with or relate to some but not all characters in the game. As game characters are presented in a predetermined sequence, some users may never witness a character that they share commonalities with, reducing the potential impact of the in-game narrative for certain users based on their progress in the game. The representation of different sexualities and genders was well received by participants as they acknowledged that it could help to address misconceptions that HIV only effects MSM, indicating that the design flaw was the delivery of the narratives rather than the inclusion of multiple character types.

#### Gameplay Perceived as Enjoyable but Potentially Limited in Appeal

Despite a positive response from the participants, in which gameplay was described as “enjoyable, addictive, and challenging,” there were indications that the game would have a limited appeal. In the first instance, participants had very low expectations for the game and expressed surprise at playing a game comparable with commercial games on the app stores. In 1 FGD, participants identified 2 categories of gamers, casual and hardcore, and discussed which category the game would appeal to and why. A casual gamer was perceived to be someone who occasionally engaged with digital games as a form of distraction, whereas a hardcore gamer’s life would revolve around digital games. Most agreed that the game would have little to no appeal for hardcore gamers. For casual gamers, the consensus was that the game would be appealing but required an effective marketing and deployment strategy to overcome competition from similar games. Offline gameplay was perceived to be a strong motivator, regardless of gamer type, as it would enable players to alleviate boredom when they were unable to access features on their devices that required an internet connection. Marketing *Battle in the Blood* as a game about HIV was perceived to have 2 effects on the appeal; in most cases, it would arouse curiosity or tap into a desire to learn, whereas in some cases, it could be off-putting. Participants felt that the game’s marketing should also be targeted toward parents and should offer information on the age appropriateness of the content.

### Phase 3: Game Analytics

Game analytics reported 3325 unique device installs globally during a 12-month period. The game received an average of 10 installs per day. Installs peaked during active and incentivized marketing events; the maximum number of installs in 1 day was 367 at the time of the World AIDS Day launch event in Davao.

Unity Analytics provided a more accurate report of game uptake and reach than the app store services because of the common practice of using third-party apps, such as SHAREit, to access the game via Bluetooth and avoid data costs. Installs via this method would not have been captured by the analytics services provided by the Apple App Store or Google Play at the time of the study.

#### Regional Game Uptake

**Figure 6:**
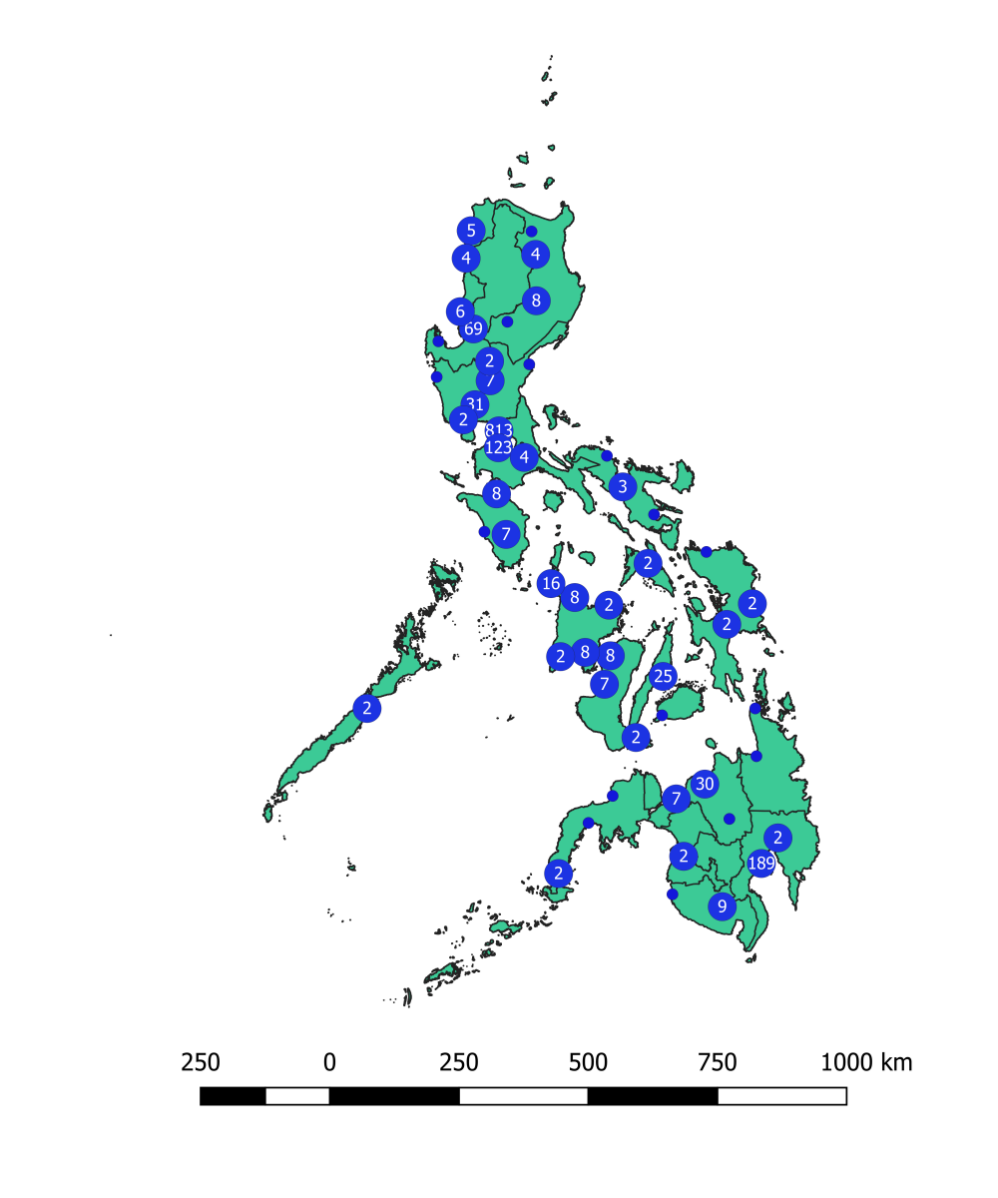
Cluster point map of the location of Battle in the Blood installations in the Philippines. Points clustered at 4mm. Number in circle represents total number of users which installed Battle in the Blood in that location. Total number of user records from the game analytics that contain geolocation data and have played the game in the Philippines=1441. Region level shape file from Humanitarian Data Exchange.

Geolocation data were collected for at least one play session from 50.77% (1688/3325) of users. Of the users from whom geolocation data were collected, 85.36% (1441/1688) were located in the Philippines (Figure 6). Game use was concentrated in urbanized areas. Uptake of the game was highest in the National Capital Region and Davao Region. Furthermore, 54.01% (1796/3325) of the users reported their age, gender, and gender of sexual partners; of these users, 28.06% (504/1796) reported as being sexually active MSM. Within the sexually active MSM category, 47.8% (241/504) reported their age as between 25 and 34 years, 26.1% (132/504) as between 20 and 24 years, and 7.1% (36/504) as between 10 and 19 years.

Overall, 2 factors are believed to have contributed to the substantially higher uptake among target users in the regions of Davao and Manila. First, several promotional exhibitions for the game were held by project staff in these regions. The exhibitions often included prize giveaways for those who downloaded the game, indicating a dependency on these types of active and incentivized marketing events. Self-reported data indicated that marketing events targeting adolescent users were potentially lacking. Second, user-centered design activities were conducted in these regions with influencers in both the gaming and HIV community, and it is likely that they also played a role in promoting the game.

#### Low Rates of Game Completion

As of December 1, 2018, 14.98% (498/3325) of users were reported to have completed level 45 and 4.00% (133/3325) were reported to have completed level 90, the last level in the game. Factors believed to have contributed to the reported low rates of game completion are the gameplay being perceived as repetitive and having limited appeal and missing data because of the provision of offline gameplay.

Additional factors impacting uptake and progression will be explored further through interviews with end users and follow-up interviews with participants involved in the design process.

#### Identification of Access Issue on Android Devices

Game analytics and app store reviews were monitored throughout the 12-month period. Monitoring activities supported the identification of an access issue on Android devices, which was related to app permissions managed by the Google Play store. A fix was launched on November 5, 2018. Between October 5, 2018, and November 4, 2018, the average number of daily active users was 16, and this increased to 40 in the month following the fix.

## Discussion

### Establishing Design Solutions Through User-Centered Design

User-centered design methods supported the identification of contextual requirements for the game [22]. Qualitative methods provided a deep understanding of the important factors that both motivated and enabled gameplay, which are likely to have contributed to the reported uptake and use of the game. The human scatter graph method and game pitches enabled participants to reflect on their views and the views of others, which, in turn, ignited valuable discussion on the qualities the game required to both be appealing and deliver persuasive health messages. Although findings from phase 1 influenced the creative direction of the game, each design choice made by the development team was prefixed with a discussion on what was feasible within the predetermined budget and development time frame. For example, during phase 1, an action RPG in which the player could control their character’s strength and traits was found to be highly desirable, but adequate resources were not available to implement a stable and balanced underlying mathematical model for this feature. In this case, the design workshops supported the identification of a wicked problem, the solution for which was informed by the experience and best judgment of the game’s coproducers [19]. The reported low rates of game completion indicated that optimal design solutions were not achieved by the game’s producers.

### User-Centered Design and Community Adoption

Results from the game analytics indicated that users’ involvement in the game development process had a positive impact on uptake among target users. However, the extent of that impact cannot be determined from the available data. For example, although unique device installs were substantially higher in regions where user-centered design activities took place, the proportion of installs credited to such activities cannot be determined, especially with the presence of targeted and incentivized promotional events in those regions. Further studies are recommended to explore the correlation between user involvement and community adoption of games for health. The way in which target users involved in the design are credited may also be an important factor to consider.

### Narratives in Games for Health

Storytelling has long been established as an effective means of attitude and behavior change [17], and this was reflected in the findings during the user-centered design process. This also highlights the importance of having an experienced narrative designer as part of the development team to work in partnership with the subject domain and behavioral experts. Assessment of the game’s acceptability found that mobile game design that accommodates typical user behavior is not always conducive to effective storytelling. To be suitable for use in a public space, the animated stories had to be highly captivating to retain focus while also allowing the player to disengage without missing vital information. Although *Battle in the Blood* made some headway in delivering a narrative that was accessible and accommodated typical user behavior, a lack of interaction, an obscure link to the gameplay, delivery through linear episodes, and limited character identification may have rendered the animated stories ineffective in generating new knowledge and perceptions when the game is played outside a facilitated gameplay session. These design flaws could be addressed through the inclusion of additional branching narratives driven by player choice, forming a stronger link between the gameplay and cause and effect sequences in the narrative, inclusion of stories based on true events, allowing players to select narratives they are interested in, and facilitating group gameplay sessions where players are encouraged to discuss their interpretation of the narratives as part of the game’s distribution strategy.

### Multiple Data Collection Methods to Mediate Biases

Telemetry and geolocation data are widely used in the mobile games industry to improve app revenue. There are countless articles ranking key performance indicators by their value to app developers. In response to demand, a range of services are now available that process large volumes of data into actionable information with very low setup costs. In the case of the Unity Analytics service (version 2017.1), standard metrics can be obtained by toggling a switch in the Unity game engine. Although generating income may not be a primary goal, it could be argued that analytical services that capture data during gameplay are currently underutilized in games for health development and evaluation [29]. In contrast to FGDs, telemetry and geolocation data collected through the game are not biased by the presence of a researcher; however, emotive responses to gameplay cannot be captured this way. Thus, it is important not to focus solely on 1 data collection technique but rather to triangulate results from a range of different data sources to mitigate bias while providing a more complete picture of game performance.

This study explored the effect of user involvement on health intervention acceptability and community adoption and described in detail the methods used. Further evaluation of the game has been conducted to explore the game’s effect on knowledge, attitudes, and HIV service use among target users, the results of which will be published in a separate paper.

### Limitations

As with all qualitative research, findings cannot be applied to the wider population with certainty. Perceptions from aged under 18 years and subjects outside Manila and Davao were not captured. Although the project team failed to recruit TGW during phases 1 and 2 of the game development process, they were included during the formative research stage, which informed the conceptual framework for the game (Figure 1). Feedback on how the transgender character in the game was portrayed was provided by TGW known to the research team in the Philippines in an informal capacity.

The authors note that the recruitment strategy and use of FGDs is likely to have resulted in a bias toward participants less encumbered by stigma toward their sexuality or HIV status. Given that recruitment advertisements stated that participants would be inputting into the design of an HIV advocacy game, this may have also created a bias toward individuals with a vested interest in health advocacy and game design.

The timing for phase 2 protype testing was not ideal, which is believed to have impacted the changes implemented in the game design.

Certain terms used by the participants did not have a direct English translation, which, at times, led to ambiguity in the data during the translation process.

### Conclusions

By involving users from the outset, the development team was guided toward narratives that were shown to be relatable and understandable and to gameplay that provided enjoyment. The resulting product is a game that is accessible, simple, and entertaining because of universally recognized game mechanics, small file size, and offline gameplay. At a minimum, games for health should involve target users in early stages of the design process as a relatively cost-effective method of cataloging important contextual requirements and user preferences. If target users are to be tasked with developing design solutions, then interaction with the development team must go beyond FGDs. For the target user to be a valued member of the game development team, they must have adequate expertise in design, a shared goal, and be properly credited and compensated for their contributions [22]. Increased reporting of design approaches and stronger collaboration among health professionals, the entertainment games industry, and end users will support the games for health industry as it matures. Restructuring projects to involve users before determining the development budget will enable design choices to be driven more by user requirements and less by what is feasible.

## Appendix

Multimedia Appendix 1 Summary of phase 1 design process.

Multimedia Appendix 2 Battle in the Blood game design document v0.15.

## Abbreviations

ARV: antiretroviral
FGD: focus group discussion
MSM: males who have sex with males
RPG: role-playing game
TGW: transgender women

## References

[1] The Joint United Nations Programme on HIV/AIDS.

[2] Bumanglag KC (2018). Enhancing HIV/AIDS surveillance in the Philippines to ensure the transgender population's visibility. Am J Public Health.

[3] (2015). Department of Health website.

[4] (2016). Department of Health website.

[5] (2018). AIDS Data Hub.

[6] Wong VJ, Murray KR, Phelps BR, Vermund SH, McCarraher DR (2017). Adolescents, young people, and the 90-90-90 goals: a call to improve HIV testing and linkage to treatment. AIDS.

[7] Adia A, Bermudez A, Callahan M, Hernandez L, Imperial R, Operario D (2018). 'An Evil Lurking Behind You': drivers, experiences, and consequences of HIV-related stigma among men who have sex with men with HIV in Manila, Philippines. AIDS Educ Prev.

[8] van Wijngaarden Jan W, Ching AD, Settle E, van Griensven F, Cruz RC, Newman PA (2018). 'I am not promiscuous enough!': exploring the low uptake of HIV testing by gay men and other men who have sex with men in Metro Manila, Philippines. PLoS One.

[9] Canoy NA, Ofreneo MA (2017). Struggling to care: a discursive-material analysis of negotiating agency among HIV-positive MSM. Health (London).

[10] Burki T (2017). HIV in the Philippines. Lancet Infect Dis.

[11] Koirala S, Deuba K, Nampaisan O, Marrone G, Ekström AM, CAT-S group (2017). Facilitators and barriers for retention in HIV care between testing and treatment in Asia-A study in Bangladesh, Indonesia, Lao, Nepal, Pakistan, Philippines and Vietnam. PLoS One.

[12] (2018). KPMG Global.

[13] (2017). Newzoo.

[14] Hightow-Weidman LB, Muessig KE, Bauermeister J, Zhang C, LeGrand S (2015). Youth, technology, and HIV: recent advances and future directions. Curr HIV/AIDS Rep.

[15] DeSmet A, van Ryckeghem D, Compernolle S, Baranowski T, Thompson D, Crombez G, Poels K, van Lippevelde W, Bastiaensens S, van Cleemput K, Vandebosch H, de Bourdeaudhuij I (2014). A meta-analysis of serious digital games for healthy lifestyle promotion. Prev Med.

[16] Prensky M (2001). Fun, play and games: what makes games engaging. Digital Game-Based Learning.

[17] Shen F, Han J (2014). Effectiveness of entertainment education in communicating health information: a systematic review. Asian J Commun.

[18] Kort YA, IJsselsteijn WA, Poels K (2007). Digital games as social presence technology : development of the Social Presence in Gaming Questionnaire (SPGQ). Presence 2007 : the 10th Annual International Workshop on Presence.

[19] Rittel HW, Webber MM (1973). Dilemmas in a general theory of planning. Policy Sci.

[20] Pagulayan R, Keeker K, Fuller T, Wixon D, Romero R, Gunn DV, Sears A, Jacko JA (2007). User-centered design in games. Human-Computer Interaction Handbook: Fundamentals, Evolving Technologies and Emerging Applications.

[21] Giacomin J (2014). What is human centred design?. Des J.

[22] DeSmet A, Thompson D, Baranowski T, Palmeira A, Verloigne M, de Bourdeaudhuij I (2016). Is participatory design associated with the effectiveness of serious digital games for healthy lifestyle promotion? A meta-analysis. J Med Internet Res.

[23] Mayer I, Bekebrede G, Harteveld C, Warmelink H, Zhou Q, van Ruijven T, Lo J, Kortmann R, Wenzler I (2014). The research and evaluation of serious games: toward a comprehensive methodology. Br J Educ Technol.

[24] Conner M, Steptoe A (2010). Cognitive determinants of health behavior. Handbook Of Behavioral Medicine: Methods And Applications.

[25] Jessen S, Mirkovic J, Ruland CM (2018). Creating gameful design in mHealth: a participatory co-design approach. JMIR Mhealth Uhealth.

[26] Korhonen H, Koivisto EM (2007). Playability Heuristics for Mobile Multi-player Games. Proceedings of the 2nd international conference on Digital interactive media in entertainment and arts.

[27] Smith J, Firth J (2011). Qualitative data analysis: the framework approach. Nurse Res.

[28] Sekhon M, Cartwright M, Francis JJ (2017). Acceptability of healthcare interventions: an overview of reviews and development of a theoretical framework. BMC Health Serv Res.

[29] Smith SP, Blackmore K, Nesbitt K, Loh CS, Sheng Y, Ifenthaler D (2015). A meta-analysis of data collection in serious games research. Serious Games Analytics: Methodologies for Performance Measurement, Assessment, and Improvement.

