# Supplementary material for: Development of a Mobile Game to Influence Behavior Determinants of HIV Service Uptake Among Key Populations in the Philippines: User-Centered Design Process
Source: JMIR Serious Games. 2019 Dec 20;7(4):e13695. doi: 10.2196/13695 (PMC6942189; doi:10.2196/13695)
Supplement: Multimedia Appendix 1 [file games_v7i4e13695_app1.docx]

| Summary of Phase One Design Process | | | |
| --- | --- | --- | --- |
| **Structural Element** | **Desired Game Feature** | **Design Choice** | **Design Considerations** |
| Gameplay | Role playing game (RPG) - To control the appearance, strength and traits of a central game character | Limited RPG features; players select from eye, hair and outfit options to customise the visual appearance of the game’s protagonist. Every player unit has the same health, attack and defence capacity. | Balancing a game is the process of tuning game rules with the goal of preventing any component being too powerful or weak, rendering the game too easy or hard or making certain strategies or game objects obsolete. The importance of balancing becomes more pronounced in RPGs when the player has control over the characters strength and traits. Limiting the number of variable considerations in a game’s design significantly reduced the complexity and amount of time required to develop the game, ensuring it remained in scope with available resources. |
|  | Intuitive controls | Match-3 game mechanic; chosen due to its universally recognised game controls and ideal fit with mobile devices. | The match-3 game mechanic can be blended with traditional turn-based combat RPG elements, where players manage their resources in combat by matching icons on a puzzle board. |
| Feedback and reward | Bitesize gameplay – where players can play for a few minutes at a time and feel they have progressed | Linear level progression; where initial levels can be passed with ease and later levels provide challenge to ensure level completion continues to feel rewarding. Time required to complete a level is restricted by limiting the number of available moves. | By limiting the number of available moves, players that deploy strategic thinking are rewarded. Additional challenge modes, such as time challenge levels, would create better gameplay variety but was believed to have no direct benefit to the educational component of the game and therefore not a priority. |
| Conflict, challenge and competition | No in-app purchases that provides unfair advantage to other players | All content in the game is free. | As development costs were covered by project funding, and this was a proof of concept study, there was no incentive to generate revenue from the game. |
| Social features | Cooperative gameplay – perceived to provide benefit to the games appeal and persuasiveness of the health messages. | Battle in the Blood leader-board ranking players on their total score. | Due to known issues around internet connectivity in the Philippines, and no available resources to maintain and police the online community, social features were limited to a leader board. |
| Narrative | To play the hero | Players take on the role of the game’s protagonist, who enters the blood stream in an antiretroviral pill capsule and fights anthropomorphic viruses, bacteria and cancer cells using a weaponised mech-suit.  Short animated stories are used to introduce the player to the characters they are helping while giving context to the increasing difficulty and enemy unit type. | User testing is required to assess if HIV treatment is interpreted as the hero of the story. |
|  | To be transparent with the HIV content but not too overt | Include references to HIV throughout the game but avoid displaying medical terms in large font. | Difficult to determine if references to HIV are appropriate until extensive user testing has been conducted; too overt and the tone of the game may be perceived as off-putting or condescending; too obscure and the health messages may not be clear. |
|  | Driven by player choice | In the game’s final mission, the player is introduced to a character with acquired immunodeficiency syndrome (AIDS) in critical condition, it is revealed he has never been tested for HIV, treatment fails and the character dies. Throughout the game the player is awarded with fragments. These combine to form an anting-anting, a traditional Filipino charm believed to have magical powers. This amulet allows the player to travel back in time and change how the story ends for this character by encouraging him to undergo a HIV test. | Narratives require structure with believable character interactions and predetermined cause and effect sequences.  The only way to maintain narrative structure is to remove or severely limit the player's ability to affect the sequence of events. Any choice given to the player within the narratives must feel like it has genuine consequence especially if the intended purpose is to communicate the positive outcomes of a particular behaviour. |
|  | Tell the story through visuals with minimal text | Stories in the game are told using a dynamic 2D manga comic book style. Each episode has a 30 second run time and text is restricted to short lines of dialog. | The more images and animations the game contains the greater the overall file size. Careful selection of images is required to ensure users can follow the story without reading the dialog. This also restricted the inclusion of branching narratives. |
| Mobility | Small file size suited to typical data plans and the internal storage space of popular devices in the Philippines | The game is 53.4MB on Android and 70MB on iOS devices. | There are a number of techniques within the Unity game engine that can be deployed to reduce the file size without significantly impacting the quality of the graphics, animations and sound. |
|  | Offline gameplay | The entire game can be played offline, the only online features are the leader-board and links to external webpages. | Doesn’t facilitate any co-op or multiplayer gameplay which could have impact on both the games appeal and effectiveness. |
|  | Navigation that enhances the game environment and is functional on small screens | Scrolling map with interactive buttons to navigate levels. Expandable sub-menu with icons. Inspired by games such as Candy-Crush as a tried and tested interface structure. | Use of icons for menu items optimises the screen space but can create usability issues as you are dependent on the user’s interpretation. |
