# Supplementary material for: Development of a Mobile Game to Influence Behavior Determinants of HIV Service Uptake Among Key Populations in the Philippines: User-Centered Design Process
Source: JMIR Serious Games. 2019 Dec 20;7(4):e13695. doi: 10.2196/13695 (PMC6942189; doi:10.2196/13695)
Supplement: Multimedia Appendix 2 [file games_v7i4e13695_app2.docx]

HIV G.E.T

Game Design Document

V0.15

Contents

[Update History](#_gjdgxs)

[1.0 Game Overview](#_30j0zll)

[2.0 Learning Objectives](#_1fob9te)

[2.1 The Learning Objectives](#_3znysh7)

[2.2 Analytics Tools](#_2et92p0)

[2.2.1 Gameplay Analytics](#_tyjcwt)

[2.2.2 Individual Player Reporting](#_3dy6vkm)

[3.0 Gameplay and Mechanics](#_1t3h5sf)

[3.1 Game Overview](#_4d34og8)

[3.2 Story, Setting and Character](#_2s8eyo1)

[3.3 Game Interface - Overview](#_17dp8vu)

[3.4 Visual Style](#_3rdcrjn)

[3.5 Main Game Loop](#_26in1rg)

[3.6 Scoring System & Achievements](#_lnxbz9)

[3.7 Introduction/Tutorial](#_35nkun2)

[4.0 Game Elements: Sections, Levels, Enemies & Power-Ups](#_1ksv4uv)

[4.1 Sections (Learning Objectives)](#_44sinio)

[4.2 Levels](#_2jxsxqh)

[4.3 Enemies](#_z337ya)

[4.4 Power-Ups](#_3j2qqm3)

[5.0 Game Interface - Detail](#_1y810tw)

[6.0 Technical Details](#_4i7ojhp)

[6.1 Development Environment](#_2xcytpi)

[6.2 Analytics and Tools](#_1ci93xb)

[6.3 Google Sheets and Live Data](#_3whwml4)

[6.4 Initial Distribution and Updates](#_2bn6wsx)

[7.0 Secondary Software](#_3as4poj)

[8.0 Design and Development Management](#_1pxezwc)

[9.0 Risk Analysis](#_49x2ik5)

[10.0 Localisation Plan](#_2p2csry)

[11.0 Test Plan](#_147n2zr)

[Appendix 1: Key Deliverable Dates](#_3o7alnk)

# Update History

By the very nature of it this document will have a certain amount of fluidity.

Any changes made will be detailed below.

| Date | Version | By | Description |
| --- | --- | --- | --- |
| 21/12/16 | 0.1 | BF | First draft created |
| 04/1/17 | 0.11 | BF | Updated with initial feedback from CH trip. |
| 04/01/17 | 0.12 | CH | Additional info on HIV GET project member roles added and some comments/ request for additional info. |
| 04/01/17 | 0.13 | BF | Accepted majority of CH changes, and replied to comments requesting additional info. |
| 05/01/17 | 0.14 | CH | Continued comments regarding avatar design and level enemies. |
| 19/01/2017 | 0.15 | CH | Added comments from Philippines team and comments for Bobby. |
| 15/03/2017 | 0.16 | CH | Added Key learning objectives, artwork and character design and detailed script for game animations. |
|  |  |  |  |
|  |  |  |  |
|  |  |  |  |
|  |  |  |  |

Note that at certain stages the art contained in this document will be for illustrative purposes only and will not necessarily reflect final look and feel. As production proceeds any updated art will be added.

# 1.0 Game Overview

*Title:*  *HIV G.E.T (GET)*

*Genre:* Serious Gaming

*Platform:* Mobile: iOS/Google Play, with Mac/PC Analytics Tool

*Target release date:* 30^th^ September 2017, but see below for more detail

*Gameplay:* Match 3 puzzler combined with turn-based-combat

*Target group profile (Primary):* Men who have sex with Men (MSMs) in the Philippines.

*Target group profile (Secondary):* Supporters of programmes designed to help the Primary target group; the ETCH team (mainly in connection with the Analytics Tool).

To the end user, GET is a fun puzzle/combat game with a difference – it uses HIV ‘bugs’ as enemies, and HIV prevention and management tactics as tools for the player to succeed in the game.

This game approach is used to deliver a much more important function, a series of Learning Objectives around the benefits of early, and regular, testing for HIV – specifically for MSM in the Philippines.

# 2.0 Learning Objectives

## 2.1 Primary learning objective:

To identify the benefits of early and repeat access to HIV testing.

### 2.1.2 Sub-learning objectives

*text in brackets indicate where the learning objective will predominantly feature in the game. ‘In game advert’ refers to pop up boxes or info pages in the game that include links to relevant external information. Gameplay refers to the avatars quest and game levels.

1. To know that treatment for HIV is available in the Philippines. (gameplay, section animations & in game advert)

*During the match 3 combat level, icons that the player matches will represent different treatment related items. One of which will represent ARVs. The player will be able to learn about the different icons on the match board, - here it will contain information about ARVs. Also appearing in relevant locations in the game will be links to appropriate external information on ARVs available in the Philippines. By linking to external content there is less risk of in game data becoming outdated.*

2. To know HIV treatment makes your immune system stronger and able to fight off illness, reduce the effect of the illness on quality of life and lead to a significant reduction in the risk of death if started early. (gameplay & section animation(s))

*This learning objective is directly connected to the primary learning objective. If access to treatment is the primary benefit of testing then the game must also describe the benefits of treatment. This can be demonstrated in the second half of the section animation – the first animation will describe a scenario where the patient gets tested (player then goes through 10 levels) – the second animation will describe a positive outcome with indications of treatment.*

*One way to approach the difficult topic of death would be to address this in the last section animation and 10 levels. Where the patient in the first animation demonstrates high risk behaviour – even if the player completes all 10 levels the patient will die in the second animation. As a novel idea we could allow the player to replay the last section animation and 10 levels but this time allow the player to change the choices of the patient in the first animation – creating an alternative ending where the patient survives.*

3. To know HIV treatment can reduce the viral load make the disease unlikely to transmit. (Section animations + gameplay)

*A happy discordant couple will feature in one of the section animations with a baby.*

4. To identify common HIV co-infections. (gameplay)

*This is* ***not*** *about identifying symptoms of HIV co-infections but to identify infections you will be at risk from if you are HIV positive. Levels later on in the game will include levels with different enemies i.e. a TB bacteria monster.*

5. To know that free, rapid testing with same day results is available in the Philippines. (in game advert + section animations)

*Held within the game must be links to testing information – note that this may be updated to include links to self-testing kits as well. Again by linking to external content there is less risk of the content in the game becoming quickly outdated.*

*In every scenario there will be a sign displaying free rapid testing.*

6. To know there’s a risk of HIV transmission among heterosexual couples in the Philippines even though prevalence is low (section animation)

*At least one of the patients described in the section animation should be clearly perceived as straight.*

7. To know that symptoms of HIV may not appear until long after infection. (Section animation(s)

*Section animations towards the second half of the game should describe patients who only seek testing when symptoms appear – the HIV virus monster will be difficult to defeat and co-infections will be present in the proceeding 10 levels.*

8. To know condoms are effective against HIV infection (in game advert + section animations)

*Links to external condom distributors (potential for advertising revenue). Condom use featured in some section animations.*

## 2.2 Analytics Tools

This section will be expanded to detail the functionality and use of the Analytics Tool, however its broad functionality will be as follows.

### 2.2.1 Game use data

Game use will be monitored through unity analytics. The project will focus on the following standard metrics:

- **Daily Active Users (DAU)**
- **Monthly Active Users (MAU)**
- **New Users**
- **Sticky Factor –** This is a measure of engagement, of the MAU how many enjoyed the game enough to play on a daily basis.
- **Total Sessions –** The total number of sessions played on a single day.
- **Average number of sessions per DAU –** higher average sessions indicate multiple visits by each player in the single day.
- **Total daily play time**
- **Total daily play time per active user –** the time an average user spent player the game on a single day.
- **Day 1, 7 and 30 retention –** Total number of players that played the game again 1 day, 7 days and 30 days after the first play.
- The project will develop the following custom metrics:
- **Funnel analyser per level –** a defined flow of user activity, for our game we will track user activity per level. i.e. of the active players that completed level 2 how many went on the complete level 3 and so on. This vital analytic will highlight any bottle neck points or potential game breaking bugs allowing the game developer to make updates to improve player retention. I.e. if over 70% of our active players are not proceeding past level 60 we could adjust the level to make it easier to pass.
- **Click through rate (CTR) –** For all in game links to external content the CTR will be calculated. This in the no. of clicks/ no. of impressions x 100. Proving the percentage of clicks on a link to view of the link.
- **Level score –** this will support the developers in balancing the difficulty of the game by tracking the scores player achieve per level.

### 2.2.3 IP geolocation:

It is vital for researchers to be able to map out where, within the Philippines, players are accessing the game. It is not clear if that can be achieved through exporting the deviceInfo raw data from unity analytics and running a lookup for the coordinates of the IP addresses.

###

### 2.2.4 App Store Data

Some app store data will be exported for analysis in a separate program:

· App reviews

· App ratings

· Page views

· Total downloads

### 2.2.5 Game metrics specific to learning objectives

Throughout the game a small number of pop-ups will appear asking the player a question. A power-up that can be used during gameplay will be offered as incentive to answer the question. A player can always opt out of answering the question but if they do this they will not receive the power-up. There will be two question formats presented, Boolean and Likert scales.

| **Question** | **Location in game** | **Learning Objective** |
| --- | --- | --- |
| **Are you enjoying the game?**  Yes  No  Ask me later | Level 20 -29 | **-** |
| **Did you know rapid HIV testing with same day results is available for free in the Philippines?**  Yes  No | Level 20 -29 | **4** |
| **Free HIV tests are accurate and reliable.**  Strongly disagree 1 >> Strongly agree 5 | Level 30 -39 | **4, 7** |
| **HIV can be cured?**  False  True | Level 40 -49 | **2** |
| **A HIV positive person on effective treatment can’t pass on the virus?**  True  False | Level 40 -49 | **2** |
| **Would you consider getting tested for HIV?**  Yes  Maybe  Never  Ask me later | Level 50 -69 | **7** |
| **Starting treatment for HIV as soon as possible could save your life.**  Strongly disagree 1 >> Strongly agree 5 | Level 70 - 80 | **2** |
| **I learnt something new by playing this game.**  Strongly disagree 1 >> Strongly agree 5 | Level 81 - 99 | **-** |

# 3.0 Gameplay and Mechanics

GET is designed intentionally to make use of game genres that players know, love and understand.

Taking this approach means that players will instantly ‘GET’ what the game is about – reducing the chance of frustration, and the possibility of losing players before they have really got in to the game.

This remainder of this section describes the basic gameplay and general framework along with a ’walkthrough’ of a typical play session.

## 3.1 Game Overview

The two main game genres we will be using are ‘Match 3’ as used in games such as Candy Crush and character v character combat – as used in many games, but most applicably here, Best Fiends.

The player plays the ‘Match 3’ game to get power-ups, weapons and defences which they then use to defeat Enemies in the combat section.

The game will have a total of 90 Levels, split in to a number of Sections, each including an average of 9 levels which will be grouped around the final Learning Objectives.

At the start of each Section, a short (potentially interactive) animated sequence will be played which will set the scene for the upcoming batch of Levels and will introduce the next Learning Objective.

Once a particular Section of Levels has been completed, a second short (potentially interactive) animated sequence will be played which will reinforce the Learning Objective for that Section.

## 3.2 Story, Setting and Character

The player will create an Avatar that will represent them in the game. They will be able to choose the name and visual appearance – including the gender – of the Avatar.

To engage the player, keep them playing, and enable us to work Learning Objectives in to the game there will be a narrative that will be carried through the 90 game Levels. The narrative will be progressed at the start and end of each Section.

The avatar will only feature in one animation at the start - establishing his role within the game.

‘For years I’ve been perfecting this treatment,’ - *Show mech-suit*

‘That evil virus won’t know what’s hit him!’ – *Show virus monster and co infection monsters*

‘It’s time to protect the immune system and stop the virus from infecting others,’ – *Gets into mech-suit and flies up nose into the body*

‘I only hope people seek my help, before it’s too late…’ – *Flying or walking body with blood cells floating around etc*

The Avatar chosen will feature in some of the game scenes, and ideally also in the scene-setting animations. On a technical note this means that these animations will need to be carried out real-time, using the game engine.

This does complicate the development process, but an advantage of this is that it reduces download size.

### 3.2.1 Game Levels

The avatars main role within the game is during the combat gameplay.

There is a total of 90 levels. 10 levels will represent 1 HIV positive individual. If the individual has no co-infections, then only the HIV virus will appear in the 10 levels.

The overarching quest for the avatar is to protect the immune system of 8 different HIV positive individuals with increasing difficulty. The 8^th^ individual includes 20 levels.

### 3.2.2 Anting Anting

Every 10 levels a pop up will appear informing the player they have unlocked a mythical fragment. There will be a total of 8 fragments to collect which when put together creates an anting - anting. This amulet will play a role in the last section animation.

The picture below is an example of an anting- anting – they come in a variety of shapes. A common style is the triangle shape in gold with the symbol of an eye.
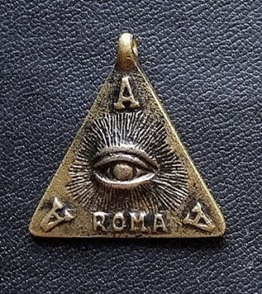


For more information on this Filipino amulet see:

<https://en.wikipedia.org/wiki/Agimat>

## 3.3 Game Interface - Overview

The game world will look like many other similar games.
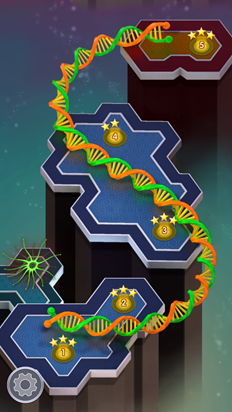


This is not down to a lack of creativity, rather it is to ensure a smooth and easy access to the game for new players.

Specific elements of the Interface are detailed in the ‘Game Interface – Detail’ section below, however broadly speaking there will be two main views.

The first view will be the Level Selection view.

This will be similar to most Match 3 games (see Candy Crush example), in that it will show a ‘road map’ of Levels. Visually these will be split into Sections grouped around the specific Learning Objectives.

Each Level that has been completed will show a ‘star rating’ encouraging replayability.

In this example ‘Sections’ are designated using colour-coded ribbon connecting the levels, for GET, given the narrative, we envisage something a lot more visually striking – perhaps being related to the particular enemy or enemies featuring in a given Section.

When the player chooses to play a level they will be taken to the main game view, which will be split screen – with the bottom showing the Match 3 game section, and the top the ‘Combat’ section.
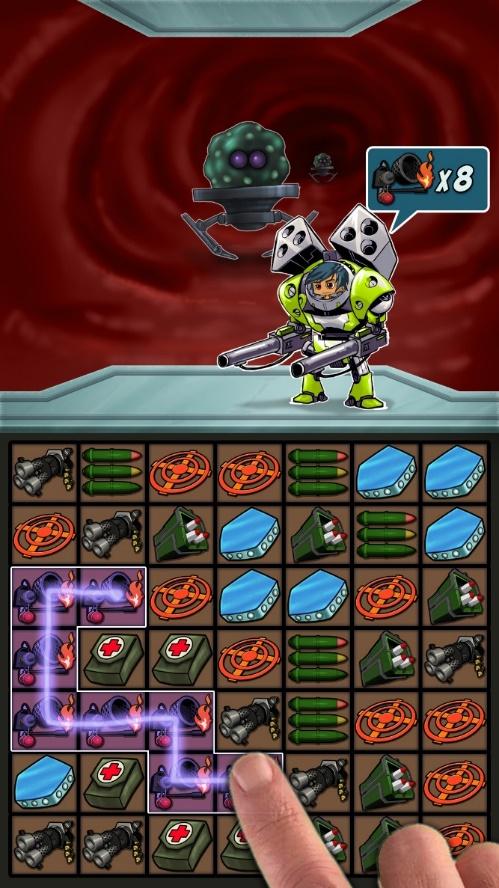


The game mechanic is described in more detail in the ‘Main Game Loop’ section below, but broadly the player has a fixed number of ‘Match 3’ moves.

Each successful move either strengthens the player or weakens the Enemy.

Once the player has used all of their moves, the Combat plays out.

At the end of the combat, they player will see a pop up detailing how well or otherwise they have done.

##

##

## 3.4 Visual Style

It is critical that the visual style chosen is one with which players are familiar and love.
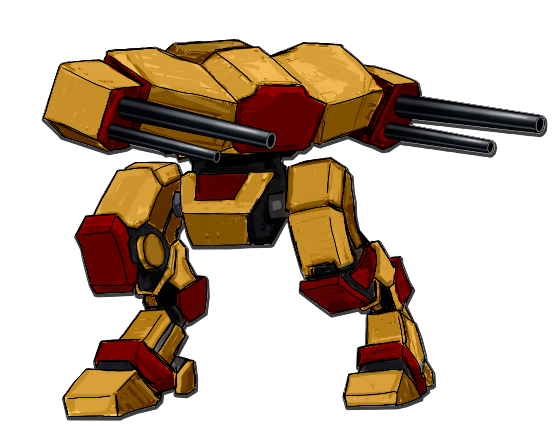


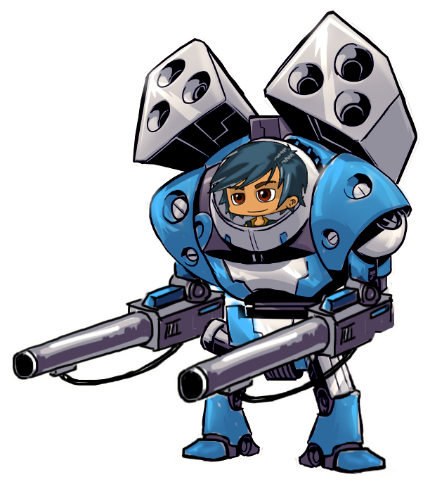


We have already created several samples for the Avatar and ‘mech suit’ that the Avatar will be wearing.


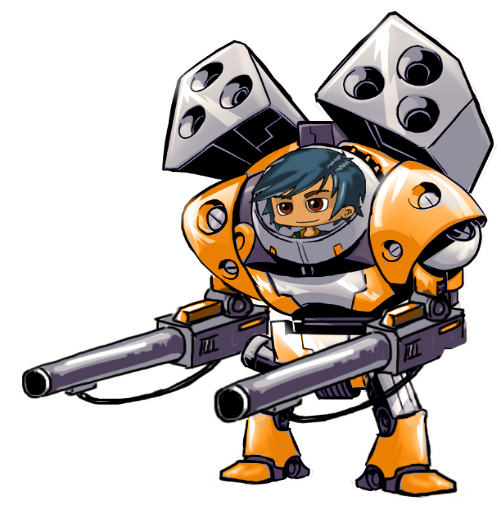


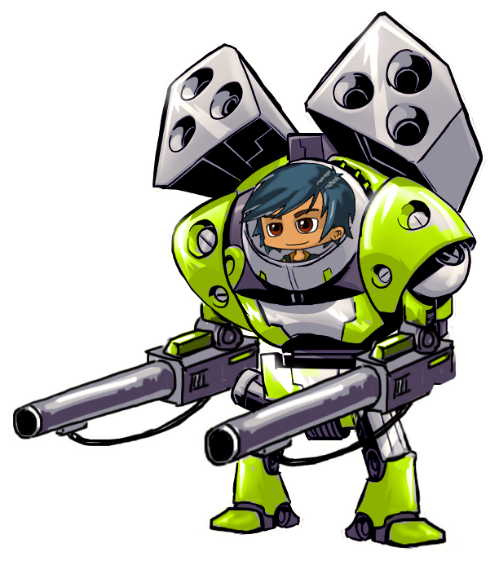


Similarly, samples showing possible Avatar variations have been created.


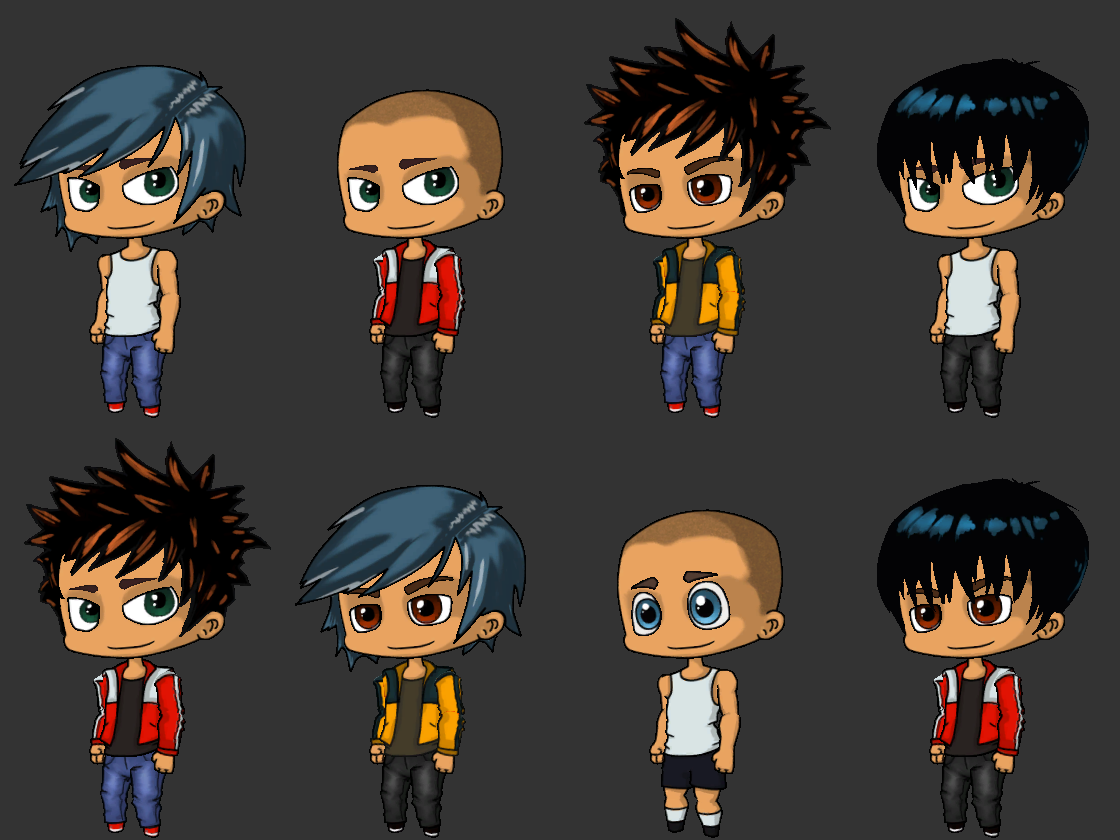


## 3.5 Main Game Loop

The main game loop is what the player goes through to complete a Level.

The player selects the Level they want to play from the Level Selection Menu. This can be any Level they have already completed, or the next uncompleted Level. The sequence is then as follows:

If this is the first Level of a new Section, an interactive animated sequence is played which introduces the Learning Objectives associated with this group. We may also at this point ask the player to share (anonymously) information on themselves.

The main game screen is now displayed.

The top part of the screen shows the current Enemy. It will have a ‘strength’ gauge labelled ‘Viral Load’ giving the player an idea of how difficult it will be to defeat. It will also show the number of combat rounds there will be this Level: the goal of each Level is to defeat the Enemy within a target number of rounds.

The player’s Avatar (inside a basic exo-skeleton) walks on to the top part of the screen. It has places for weapons, defences, special attacks etc - but none are present. The player also has a ‘strength’ gauge labelled ‘Immune System’.

The bottom part of the screen shows what appears to be a standard ‘Match 3’ puzzle game. However instead of just scoring points, or clearing the board, these objects are connected to your weapons and defences.

### 3.5.1 Match 3 Icons

5 different types; 2 defence, 3 attack. The icons are listed in order of attack and defence power i.e. no.1 would have the strongest attack.

(all images are just examples, the colours, size and shape will not be copied)
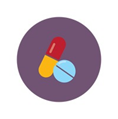


**Attack icons:**

1. Antiretroviral tablets – these come in all shapes and sizes so may be best to use a collection of 2 or 3 generic tablets for this icon so no risk of being seen to promote one over the other. <http://i-base.info/guides/category/arvs>
2. Health care – There are three ways you could represent health care.
3. Healthy living – this covers two areas; balanced diet and exercise. It’s not easy to represent this in a single icon but one option could be to use a symbol like this. (May be best not to use two heart shaped icons on the board so could affect design choice for health care.)
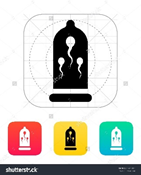


**Defence icons:**

1. Condoms – there are a number of ways to represent a condom but perhaps the clearest would be including the sperm.
2. Time – this will only be clear to the player if they read the icon descriptions. It can cover a number of areas, testing early, early access to treatment and/or taking medication on time. We have to accept that most players probably won’t read the icon descriptor but I feel it is still important to include the concept of time as a defence mechanism.

The player then has a given number of Match 3 moves to equip their Avatar’s Mech Suit. They need to decide, looking at the enemy, and the makeup of their board – what match 3 strategy they want to take to equip their Avatar – More guns? More armour? Go all out to get that ‘smart bomb’? Try to win a power-up to give another combat round?

Once the player is out of moves, the Avatar and the Enemy have a round of combat. This is automated and will take 10-20 seconds.

If the Enemy is defeated (by reducing the Viral Load to a certain level), the player wins the Level.

If the Enemy is NOT defeated, we move to the next round – the Enemy keeps any damage from previous rounds, so the idea is that if you have enough rounds you will defeat the Enemy. If though we are out of rounds before the Enemy’s energy bar is depleted, the level is lost.

Once the Enemy is defeated, or the last combat round has been played, a screen will show the player how well they did – and allow them to return to the Level select menu, replay the Level, or – if they defeated the Enemy – play the next Level.

### 3.5.2 Icon descriptors

Held within the help section of the game will be brief icon descriptors. We could also look into accessing this info from the level screen but must be careful it does not disrupt the gameplay.

Antiretroviral Medication (ARV)

HIV is treated with ARVs, these work by stopping the virus replicating in the body, allowing the immune system to repair itself and prevents further damage.

HIV positive people on effective treatment have an undetectable viral load so can’t pass on HIV.

ARVs are available in the Philippines. For more information, click the ?.

<http://www.unfpa.org.ph/index.php/resources/quick-facts/211-hiv-aids-treatment-hubs-in-the-philippines>

Health Care

Doctors and counsellors are here to provide health care and advise without judgement or discrimination.

For more information, click the ?.

<http://www.gov.ph/2016/02/12/list-doh-hiv-testing-centers/>

Healthy Living

It is vital you take care of your health, especially if you suffer from a chronic illness. Simple things such as eating a balanced diet, regular exercise and practicing safe sex is good for both your head and your heart.

Condoms

Latex condoms, used consistently and correctly, are 98-99% effective in preventing HIV infection during sex.

Time

There is no time like the present to look after your health. Early and repeat testing if you are at risk of HIV and taking medication on time is one of the greatest defences against HIV.

## 3.6 Scoring System & Achievements

As with all games, scoring and achievements are important.

The main scoring mechanic in GET will actually be Level progress, but we will also show a ‘star count’ on the Level Selection menu.

This will be a combination of the stars achieved for each completed Level – so if two player are at the same Level, they can be differentiated by how many stars each has achieved.

We will also support Apple’s Game Center and Google Play’s Game Services, and use it to maintain Leaderboards and Achievements.

The Achievement List is still to be finalised but could include the following:

- Reached Section ‘X’
- Defeated an Enemy first time
- Defeated an Enemy whilst taking no damage
- Got max stars for a Section

## 3.7 Introduction/Tutorial

In any game it is important that new players are guided through the initial phases to ensure the player does not give up in frustration if they don’t know what to do or cannot proceed at any stage.

Once all game mechanics are finalised, on-boarding will be designed and included here prior to implementation in the game.

The first level will take the player through the key game functionality. Brief tutorials will be included if any new features are added later on in the game.

# 4.0 Game Elements: Sections, Levels, Enemies & Power-Ups

There are four distinct elements which make up the game. These will be determined as game development proceeds, and this section will be regularly updated with the latest thinking. At this stage though, a brief description of each is given.

## 4.1 Section Animations (Learning Objectives)

A motion comic strip style will be utilised as it is not resource heavy and animation can be built and edited in a relatively short time frame. An example of this style can be seen here:

<https://www.youtube.com/watch?v=HRpSRVUpBEc>

The graphics should be detailed but not hyper realistic. Advised to use a manga style.

### 4.1.1 Overview

| No. | Intro | Level monsters | Ending | Learning objective |
| --- | --- | --- | --- | --- |
| 1 | Male and female together in a bar, alcohol clearly in scene> hotel room w/ do not disturb> Sat together at clinic ‘6 weeks later’> show characters holding a piece of paper one saying negative, the other saying positive but do not reveal which character is positive. | HIV virus | Start from where the last scene ended and zoom out to reveal Female is negative male is positive> counsellor giving advice on treatment for discordant couples> ‘1 year later’ couple looking happy with a baby. | 1,5,6,2 |
| 2 | Young male wakes up with another man in bed > goes straight to the clinic > non-reactive, counsellor asks him to return in 6 weeks > he returns in 7 months | HIV virus | Takes medication> at the gym > having fun with friends | 1,2 |
| 3 | male with symptomatic gonorrhoea walking in pain, looking at urinal in pain > gets tested during outreach event | HIV virus  Gonorrhoea | Counselling informing that he is all clear of gonorrhoea> second scene of him taking HIV medication> finish on happy scene | 1, 2, 3 |
| 4 | Transgender, very glamorous > sees severe looking rash > peer or friend (attractive male) advises her to come to the social hygiene clinic with them | HIV virus  Herpes virus | TG sat with service provider > TG at beauty contest | 1, 2 ,3 |
| 5 | Man sitting in office looking worried ‘what if I am, what if my boss finds out and I lose my job?!’> 3 years later, show guy looking in the mirror much thinner with a sore > reads something about confidential testing | HIV virus  Herpes virus | Takes medication, puts on tie> goes to work, boss calls him into his office> nervous he enters only to have his boss congratulate him on his report | 1,2,3,6 |
| 6 | Wife waiting for husband to return in airport> he says he’s worried she looks so much thinner than when he last saw her and seems very un-well> ‘what if I caught it from him?!’ > goes to clinic | HIV virus  Hepatitis B virus | Close up of woman in hospital, ‘I know I got this from you, but I forgive you…’> zoom out to reveal a man that is not her husband > show husband sat on plane ‘meanwhile…’ | 1,2,3 |
| 7 | Young looking male in internet café playing games> runs out of money > meets an older man outside the internet café who hands him money> ‘7 years later’ coughing and clear weight loss | HIV virus  Hepatitis B virus  TB bacteria | Young man sat with doctor ‘you were very lucky, if you had left it any longer before starting treatment this could have been a very different ending for you.’ | 1,2,3,6 |
| 8 | Man rushed into hospital, looking extremely unwell > shot of arm with distinctive tattoo> in intensive care room | HIV Virus  TB bacteria  Cancer cell | The man dies> shot of grave stone> mourners | 1,2,3 |
| 9 | The avatar reverses time to 10 years before scenario 8 using the anting anting > show the tattoo to help player identify the character as he will look different > they approach the man from scenario 8 Avatar -‘oh no I’m too late, the virus is already inside,’, the player is then given 3 options: Tell the man to get tested for HIV > tell the man to enjoy his life to the fullest while he still can > tell the man to take his medication | HIV Virus | If the player selects ‘tell the man to get tested’  10 years later…shows the man alive    If the player selects the other 2 options > repeat section animation 8 from the intensive care room scene | 1,2,3 |

### Section animation 1: Intro

Transitions between each scene are down to the lead artist. Recommended to use transitions, and motion to some of the illustrations, as used in the example below.

<https://www.youtube.com/watch?v=HRpSRVUpBEc>

Both intro and ending animations should aim for a run time of no more than 15-20 seconds.

The intro always has 5 scenes, the ending is made up of 3 scenes.

In every intro scene there needs to be a poster, sign or advert placed in the illustration reading ‘Free HIV Rapid Testing’ apart from section animation 8.

| **Text** | **Visuals** | **Reference images** |
| --- | --- | --- |
|  | Male and female characters, attractive well-dressed but not overtly wealthy. Talking to each other in a bar alcoholic drinks in hand. Flirtatious poses. |  |
|  | Couple run hand in hand through a hotel room door. |  |
| ‘I don’t normally do this on a first date…’ | Close-up of a ‘Do not disturb sign’ Could add some motion of the sign swinging on the door handle. |  |
| *6 Weeks Later* | Couple sat together in a clinic waiting room, looking nervous but supporting each other.  Include a poster displaying ‘Free Rapid Testing’ and repeat this image in all clinic scenes or scenes associated with testing in all section animations. | 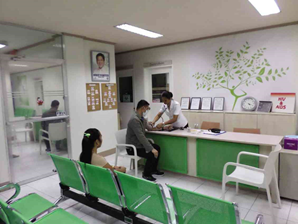Reference image from Klinika Bernardo Philippines – one of our study clinics. |
|  | Characters holding a piece of paper, one reads positive for HIV antibody one reads negative for HIV antibody but don’t show which character is which. | 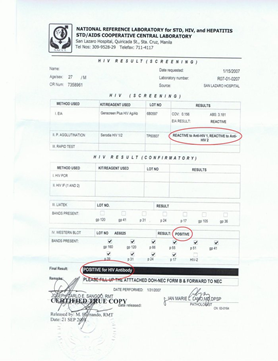Do not include any recognisable signatures or addresses from this reference. The form shown in the animation does not have to be as detailed and should avoid technical words. |

### Section animation 1: Ending

| **Text** | **Visuals** | **Reference images** |
| --- | --- | --- |
|  | Begin where the last scene ended and reveal that the female is positive and the male is negative. |  |
| ‘Thanks to treatment this doesn’t have to be the end of your relationship.’ | Couple are sat with a doctor, doctor is talking to them and has in his hand a pill bottle, on the label is ARV but doesn’t necessarily have to be legible. |  |
| 2 years later | Male and female walking on beach or shopping mall with a cute baby being affectionate with each other. |  |

### Section animation 2: Intro

| **Text** | **Visuals** | **Reference images** |
| --- | --- | --- |
|  | Attractive youthful Male wakes up in bed looking a little dishevelled wearing a t-shirt. |  |
| ‘why do I never make the cute ones wear a condom?!’ | He realises there’s another man in his bed still asleep. |  |
|  | Male sat in clinic wearing the same t-shirt as the first scene to indicate this is the same day. (can reuse background from section animation 1: intro) | 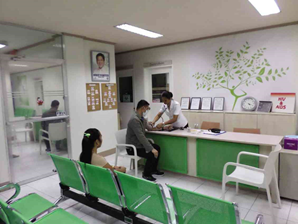 |
| ‘Your results are negative…please come back in 6 weeks as you may still be at risk’  *20 minutes later* | Male sat with a doctor talking to him. |  |
| *7 months later* | Male sat in clinic looking nervous. |  |

### Section animation 2: Ending

| **Text** | **Visuals** | **Reference images** |
| --- | --- | --- |
| ‘you should have returned sooner, you gave the virus time to become stronger and attack you’ | Male is sat with a doctor, doctor is talking to him and has in his hand a pill bottle, on the label is ARV but doesn’t necessarily have to be legible. |  |
| ‘at least I have the power to fight back now.’ | Close up of pill bottle in a hand with Antiretroviral on the label. | 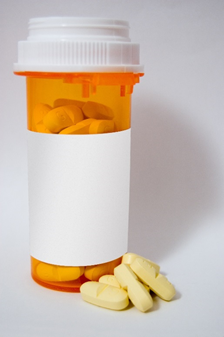 |
|  | Male at gym, lifting weights and looking happy. |  |

### Section animation 3: Intro

| **Co-infection** | | Gonorrhoea | |
| --- | --- | --- | --- |
| **Text** | **Visuals** | | **Reference images** |
|  | Male at nightclub with male friends, looks in pain. | |  |
| ‘It felt good at the time, but now I wish I’d worn a condom.’ | Stood in bathroom looking at the urinal in terror holding his crotch. | |  |
| ‘we’re offering free testing in the club tonight, interested?’ | Talking to a second male character in the nightclub, red faced and still looking in some pain. | |  |
|  | Male character sat with nurse in a private room as he takes a sample of blood using a finger prick. | | 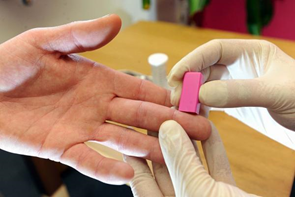 |
| ‘come to the clinic tomorrow for more tests, ok?’ | Close-up of a rapid test showing a positive result held in gloved fingers. | |  |

###

### Section animation 3: Ending

| **Text** | **Visuals** | **Reference images** |
| --- | --- | --- |
| ‘it was the gonorrhoea that was causing you pain,’  *2 weeks later* | Male is sat with a doctor, doctor is talking to him and has in his hand a pill bottle, on the label is ARV but doesn’t necessarily have to be legible. |  |
| ‘we can cure gonorrhoea and life can go on with HIV treatment.’ | Close-up of pill bottle with antiretroviral on the label. |  |
|  | Male at a beach or mall happy and with friends. |  |

### Section animation 4: Intro

| **Co-infection** | | Herpes Virus | |
| --- | --- | --- | --- |
| **Text** | **Visuals** | | **Reference images** |
|  | Female on stage in a competition with sash on. | | 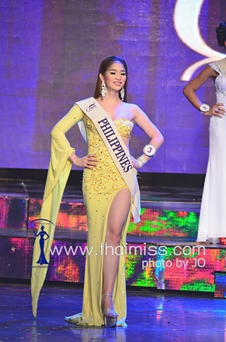 |
|  | Female still in beautiful outfit, looks like she’s in mild discomfort holding her side as she stands in the changing rooms. | | 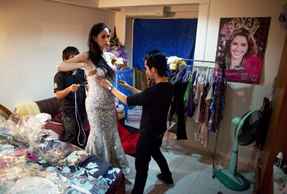 |
| *Few days later* | The female is now in pyjamas with no makeup with her top lifted revealing a rash. She’s in a different environment to the first scene, potentially a bathroom or bedroom. | | 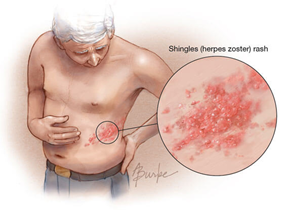 |
| ‘This could be the sign of something much worse, come to the clinic with me tomorrow’ | Female listening to a male friend in a social environment, her outfit is simple and she looks concerned. | |  |
| *the next day….*  ‘It’s reactive, we’re going to need to conduct more tests.’ | Close up of a rapid test showing a positive result held in gloved fingers. | | 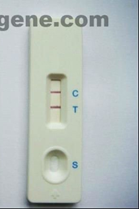 |

### Section animation 4: ending

| **Text** | **Visuals** | **Reference images** |
| --- | --- | --- |
| ‘I know this competition is important to you, but so is your medication.’ | Female sat with doctor, pill bottle in his hand. |  |
| ‘I fought to be my true gender, I can win this fight too.’  *1 year later* | Female in changing rooms taking tablets from pill bottle. (repeat environment from 2^nd^ scene in intro) |  |
| *‘*My name is Linda Viveka, 26, Philippines’ | TG introducing herself in a beauty contest | 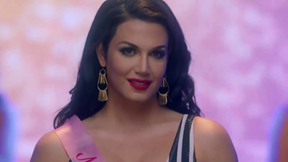 |

###

### Section animation 5: Intro

| **Co-infection** | | Herpes | |
| --- | --- | --- | --- |
| **Text** | **Visuals** | | **Reference images** |
| ‘what if I am, and what if my boss finds out I’ll lose my job.’ | Male sat at his desk, wearing a shirt and tie, on his own looking concerned in a typical office environment. | |  |
| *3 years later* | Male looking in mirror with cold sores on his lips and some weight loss. | | 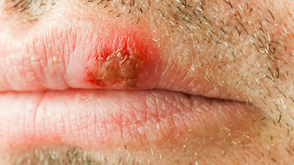 |
|  | Sat at a computer reading an article with the title Looking for Confidential Testing? | |  |
|  | Male sat in the clinic. (repeat clinic environment) | |  |
|  | Male holding a piece of paper displaying positive for HIV antibody. | | 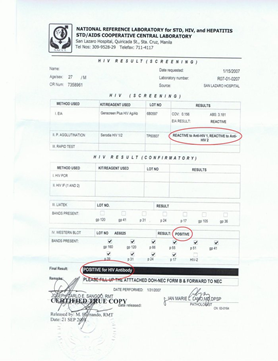 |

###

### Section animation 5: Ending

| **Co-infection** | | Herpes Virus | |
| --- | --- | --- | --- |
| **Text** | **Visuals** | | **Reference images** |
| 1 month later | Wearing a shirt and tie, looking much healthier with weight gain the male takes a tablet. | |  |
| ‘can I see you in my office?’ | In the office an older second male character (the boss) stands behind him, he has a straight face. The male character looks nervous. | |  |
| ‘great job on the report, keep that up and you could have a real future here.’ | In the boss’s office both characters look much happier. | |  |

### Section animation 6: Intro

| **Co-infection** | | Hepatitis B | |
| --- | --- | --- | --- |
| **Text** | **Visuals** | | **Reference images** |
|  | Female character, looking very thin and unwell. Stood in airport arrivals. | |  |
|  | Male character sat on plane. | |  |
| ‘It’s great to see you here, but my darling wife you don’t look well.’ | Male character talking to female character at arrivals. Characters are affectionate towards each other. | |  |
| *1 month later* | Female sat in clinic looking very unwell. | |  |
| ‘We never use condoms, what if he gave it to me?’ | Female holding a piece of paper displaying positive for HIV antibody. | |  |

### Section animation 6: Ending

| **Text** | **Visuals** | **Reference images** |
| --- | --- | --- |
| ‘I know you gave this to me…but I forgive you’ | Close up of female in hospital. |  |
|  | Zoom out to show she is with another male that is clearly not her husband. They are affectionate towards each other. |  |
| *Meanwhile…*  ‘I wonder if she’s waiting for me this time?’ | Husband getting off plane. |  |

### Section animation 7: Intro

| **Co-infection** | | Hepatitis B + TB | |
| --- | --- | --- | --- |
| **Text** | **Visuals** | | **Reference images** |
|  | Young male playing game on a computer in an internet café. | |  |
|  | Male leaving the café, looking sad, pulling the material out of his empty pockets indicating he has no money left. | |  |
| *Later that day* | In a deserted alleyway an older male hands the young male money. | |  |
| *7 years later*  ‘You should have a HIV test; it may be the cause of all of this.’ | The male looks older, thinner and unwell. He is coughing. He is sat with a doctor. The doctor is talking to him.  (the doctor and his office will look significantly different to the previous section animations) | |  |
| *Later that day* | Male holding a piece of paper displaying positive for HIV antibody. | |  |

### Section animation 7: Ending

| **Text** | **Visuals** | **Reference images** |
| --- | --- | --- |
| *2 months later*  ‘You seem to be responding well to the treatment.’ | The male is sat with the doctor from the intro. The doctor is talking to him. |  |
| ‘this medication saved my life.’ | Male stood outside hospital doors (see ref of Philippine General Hospital) looking at a pill bottle. | 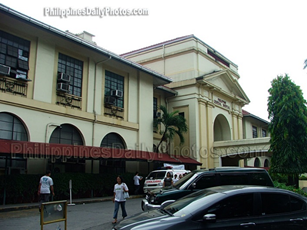 |
| ‘For someone else it may already be too late.’ | In front of the hospital the male walks past a tricycle (see ref) and sees a very sick looking male slumped in the sidecar. Description of this character’s appearance is in section animation 8: intro. The driver is dismounting the motorbike. | 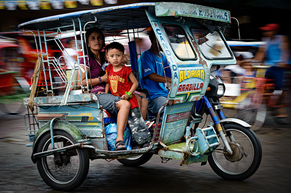 |

### Section animation 8: Intro

| **Co-infection** | | Cancer cell + TB | |
| --- | --- | --- | --- |
| **Text** | **Visuals** | | **Reference images** |
| ‘This man needs a doctor, now!’ | Male character slumped in sidecar of tricycle, looks very thin. The driver is shouting. | |  |
|  | The male is lifted out of the sidecar by two nurses. | |  |
|  | The male is now being pushed inside the hospital in a wheelchair still slumped. There are dark purple lesions on his face and arms (see ref) this is Kaposi sarcoma. There is a distinctive tattoo clearly visible on his arm. | | 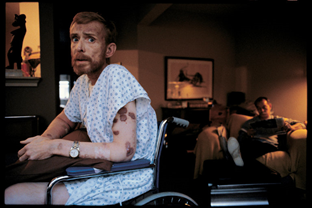 |
| ‘Has he ever been tested for HIV?’ | Male in hospital bed with eyes closed. Heart monitor and saline drip visible. Doctor and second male character are stood either side of the bed, the doctor is talking. | | 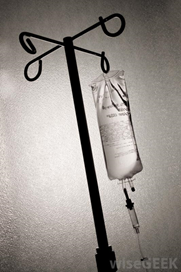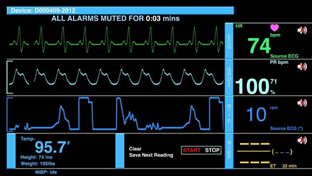 |
| ‘No.’ | Close up of second male characters’ face responding and visibly upset. | |  |

### Section animation 8: Ending

| **Text** | **Visuals** | **Reference images** |
| --- | --- | --- |
|  | Male in hospital bed by himself, eyes closed. Heart monitor and saline drip visible. |  |
|  | Close up of heart monitor as it flat lines. | <https://www.youtube.com/watch?v=Q_gzl_E7jmw>  (skip to 1m:30s) |
|  | Shot of gravestone. Etched into the stone:  ‘Bruce Wayne 1987 – 2017’ |  |

### Section animation 9: Intro

| **Co-infection** | | Cancer cell + TB | |
| --- | --- | --- | --- |
| **Text** | **Visuals** | | **Reference images** |
| ‘I now understand my true mission.’ | The 8 fragments of the anting anting glow and rise up from the avatar’s hands. | |  |
| ‘I have one last chance to save his life.’ | Close up of the fragments joined together creating the complete amulet. The amulet appears as a strong magical power, could also show clocks indicating time is reversing. | | 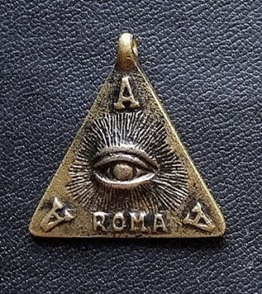 |
| *10 years earlier*  ‘No! I’m too late, the virus is inside and he’s stronger than ever.’ | The male from section animation 8 is walking along with his friend who was at the hospital, he looks younger, attractive and healthy. The tattoo on his arm is visible. The avatar is floating to the side of his head and hasn’t been noticed. The avatar is very small compared to the two male characters but still visible to the player, the avatar is speaking. | |  |
|  | Zoom into the avatar by the male character’s ear whispering. The animation pauses and the following question pops up:  Do you  a) Tell the man to live the next 10 years to the fullest  b) Tell the man to take his medication  c) Tell the man to get tested | |  |
|  | Man looks confused, has turned his head to where the avatar was but is no longer there. | |  |

### Section animation 9: Ending

Play this ending if player selects option c). If player selects the other 2 options, then repeat section animation 8: ending.

| **Text** | **Visuals** | **Reference images** |
| --- | --- | --- |
| *10 years later*  ‘If you had only got tested, maybe you would still be here today.’ | Show the back of a gravestone, a man is stood in front of the gravestone facing it. He is not recognisable (only part of him shows or he is silhouetted) |  |
|  | The male is clearly in shot, it is the man from the intro animation, the tattoo is visible. He looks older but healthy. He has a pill bottle in his hand. |  |
| ‘getting tested saved my life, it could have saved yours.’ | Close up of pill bottle in hand. Antiretroviral on the label. |  |

## 4.2 Levels

There will be 90 Levels in the game.

Level progression will be based on two criteria – general gameplay difficulty, as well as integrating with the Learning Objective narrative flow.

Developing 90 challenging and fun levels is no small task, doing so where game elements must also fit with Learning Objectives will be even more difficult.

We envisage a key part of development will be creation of a ‘Level Editor’ so that levels can be created, tested and amended quickly.

We will also build ‘evaluation’ tools in to the beta versions of the game so we can get feedback from testers in as efficient a manner as possible.

## 4.3 Enemies

Functionally all enemies will behave the same way.

They will have a Viral Load (their defence strength) and they will have attacks.

Attacks will be ‘projectile’ based, so the Enemy will animate and ‘fire’ at the player’s Avatar.

What will vary for each enemy will be:

- Starting Viral Load: how hard it is to defeat
- Visual Appearance: what it looks like, ie, what type of enemy
- Special Effects for Attack: lightning bolt, trail of fire etc
- Frequency of Attack: recharge time between attack
- Power of Attack: damage each attack does

These parameters should give an understandable set of Enemy variations, whilst also providing plenty of variety.

Enemies will be depictions of the virus or bacteria with facial features. Giant microbes are a good place for inspiration but are a little too cute for our monsters, we want them to look evil.

<http://www.giantmicrobes.com/uk/>

We are currently proposing 6 different enemies. Where each enemy will appear in the 90 levels is detailed under section animation scripts.

**HIV Virus**

The primary enemy is the HIV virus monster and will make an appearance in every batch of 10 levels.

**Tuberculosis bacteria**

TB is the leading killer of HIV positive people so this needs to be a very tough enemy. The TB bacteria will only appear in the later missions.

**Cancer cell**

Cancer is a disease caused by abnormal cell growth, so our enemy will be a mutated and monstrous looking cell. Again this will be one of the toughest enemies to defeat.

**Hepatitis B**

This is a virus so will be a similar shape to the HIV virus. Hep B is a more stable virus than HIV – in that it can survive on surfaces for longer – this could be represented by the virus wearing armour or having a tough outer looking shell.

**Gonorrhoea**

Although a bacterium like TB, Gonorrhoea has a very distinctive shape and is referred to as diplococci which is two spherical shapes joined together.

**Herpes Virus**

We’ll be using 1 virus monster to represent two viruses as they belong to the same family, Herpes simplex 1 and 2 (better known as cold sores) and *Herpes Zoster* (sometimes known as shingles). One of the key features of this virus are the glycoprotein spikes so could be represented with a spikey virus monster.

## 4.4 Power-Ups

Whenever the player does a ‘Match 3’ move they will win the ‘Power-Up’ associated with the icons they have connected. There will be three different categories of these, some examples of each are given below:

*Equip Player:*

Add weapon: adds to player’s arsenal. There will be 4 different weapon types.

Add armour: adds to player’s defences. There will be 4 different defence types.

*Global Player Aid:*

Damage multiplier: multiplies ALL player attacks.

Damage reducer: reduces damage from ALL Enemy attacks.

*Pre-damage Enemy:*

These can reduce the enemies Viral Load immediately, ie, even before the combat has started

*Specials: (Note these will be collected by doing ‘Special’ connections)*

- Slow enemy: drops Enemy’s attack frequency.
- Extra Round: adds an extra round of combat.
- Extra Life: if the player’s Treatment Power goes to zero, this will reset it to full.

# 5.0 Game Interface - Detail

This section will include images and descriptions of all key user interface elements as and when each is finalised.

# 6.0 Technical Details

Resistance will be built using the game engine Unity 3D.

## 6.1 Development Environment

Unity 3D is the most widely used game engine in the industry which makes this a good choice for a number of reasons:

- First class support from company.
- Huge and active community of users means strong secondary support.
- Thousands of Unity 3D titles ‘in the field’ means high likelihood of strong compatibility across a wide range of hardware and software. This is particularly important when one target platform is Android.
- Should future work/updates be required and the original developer is not involved, the large number of potential professional developers using Unity 3D provides future proofing.
- Unity 3D is the most multi-platform engine on the market, so should other platforms be required in the future the porting will be simpler.

During development the game project will use an SVN server source control system hosted by Amazon. A welcome side effect of this approach is that the game project is always automatically backed up in a secure offsite location.

## 6.2 Analytics and Tools

There are many analytics solutions available, however we propose to use the latest analytics built in to Unity 3D. These provide simple, yet powerful, analytics on how a game is being used. They enable us to garner statistics about what our players are doing in the game.

More information can be found here: <https://unity3d.com/services/analytics>

These analytics focus on the user group as a whole. We will also provide the ability to analyse the behaviour of individual players.

At various points players are asked if they want to share information on themselves. The intention is that this data will be stored on a server in a common file format (Excel or Access) so that industry standard tools can be used to interrogate it.

## 6.3 Google Sheets and Live Data

Google Sheets are free and can be used by anyone with an internet browser on their hardware – so they should be available to all of our players and their managers.

We will use Google Sheets for both back end data and, possibly, individual player reporting.

The way the back end data will work is that, wherever possible, any ‘fixed’ data in the game is taken from a Google Sheet rather than hardcoded in to the game.

This has two advantages. The first is that non-coders can view/edit this data easily during the development phase. The second is that we can use a ‘live update’ system. Effectively this lets us change certain things about how the game works WITHOUT needing to distribute a new version of the game.

If for example, on looking at analytics after launch, we realise that there is a problem with the difficulty of level 6 because the Enemy is too strong, then - in the appropriate Google Sheet - we can either reduce the strength of that enemy, increase the number of rounds, or frequency of ‘special power ups. We then package these, add them to our server – then the next time a player starts the game, it will use these new values.

### 6.3.1 Section animations text management

As Google Sheets will also be used to store all text in the game, it will give us flexibility there too.

Text will be kept to a minimum with most focus placed on the visuals. Each animation will be split into coded scenes.

This data will be held in google sheets allowing text to be edited outside of the game development engine. Allowing designated project members to edit and review game text.

There are limitations to what can be edited after the section animation has been built. For example, it would not be possible to add another scene. Any changes to the visuals will have to be conducted by the game developer. Any major changes to the visuals after sign off on the section animation storyboards will be identified as a change request and may incur additional costs.

The game developer will have to release an update if text in the google sheet is edited after the games release for the new game data to appear in the game. Updates after releasing are included in the contractual agreement with the game developers.

## 6.4 Initial Distribution and Updates

The final game will be publically released via Apple’s App Store and Google Play.

Prior to that alpha and beta builds will be distributed using either TestFlight or, preferably for ease/speed, the Google Play Beta System.

We will also build ‘time locking’ in to these pre-release builds so that if they are pirated, they will not work after a certain period.

Any updates that cannot be done using ‘Live Data’ will be submitted as normal through the Apple and Google update system.

# 7.0 Secondary Software

Tools used during the game development:

- Unity3D: game development
- Photoshop: 2D art for GUI and texture creation
- 3DS Max: 3D art and animation
- Unity Analytics: game analytics and reporting
- Google Sheets: game data management and individual user data reporting; ‘live data’ game updates.

# 8.0 Design and Development Management

Communication is a key element of any game development project, but even more so when three teams with important, but different, skill sets are working together.

Ensuring the game development team properly understand all major aspects of the environment to be simulated is crucial.

It is also important to ensure that the LSTM team and, where appropriate, experts in the Philippines, understand what is happening at each stage of development to a level where they are able to provide feedback and guidance to ensure the final game meets their requirements.

To do this the best approach is to make sure there is regular communication – both structured and ad hoc as and when required.

A weekly Skype meeting will take place to cover all relevant development issues. Minutes will include a series of action points to be carried out that week. This regular meeting should ensure no major slippage issues occur without the team seeing this is happening and being able to discuss steps to remedy the issue.

It is envisaged that shortly after each of the key interim playable deliverables (technical prototype, alpha and beta) that a round table meeting of the relevant team members will be held.

A number of software tools will be used to assist management of the development:

- Skype: to enable the weekly, and any other, ‘offsite’ meetings.
- Balsamiq: to do quick 2D prototyping of any new aspects of the game design.
- Trello: a task management system, particularly suited to visual iteration.
- FastIssueTrack: a more detailed bug/issue tracking system for handling specific reports and iteration.

# 9.0 Risk Analysis

We see no technical risks in any aspect of the project.

All art and code related functions are in areas where we have a great deal of experience.

Possible risk areas are as follows:

*Low Spec of Target (Android) Devices in Philippines*

It is likely some of the target users will have low end devices which, whilst they will play games, may not have the power to play graphically rich games.

To mitigate this, the very game genre chosen, is one that can normally be run on lower powered devices.

Further, we intend to create ‘Level of Detail’ versions of the player and Enemy characters. This should mean on lower spec devices an acceptable frame rate can be maintained.

*Game Size*

Many players are uncomfortable downloading games over a certain size – partly because of bandwidth costs/availability, but also due to the footprint on the device.

Again, the game genre chosen helps to mitigate this.

Also, the instructional/narrative video sequences will be replayed using real time rendering in the game engine which will result in significant footprint savings.

*Target Users Not Engaging In Game*

Users may just not like the game enough to play it sufficiently to cover all of the Learning Objectives.

This is a trickier one as not every person is going to like every game type, however we have made several design decisions intended to make the game attractive to as wide an audience as possible:

- We have picked a hugely popular game genre, and one which is popular with a wide range of demographic.
- We have picked a game genre that is ‘pick up and play’ by nature, and which works well in short gaming sessions.
- We are involving the target users in decisions about the visual appeal of the game.

# 10.0 Localisation Plan

All text that appears in the game will be taken from a Google Sheet.

The sheet will have a number of columns equal to 1 plus the number of languages supported. At launch the intention is for the game to ship in Filipino and English, therefore there will be three columns.

The first column will be an internal game ID. This is what the game uses when displaying a piece of text. So for example, the text on the leaderboard button ‘High Score’ would be stored in code as ‘ID_HS’. The three columns would then look like this:

| *ID* | *English* | Filipino |
| --- | --- | --- |
| ID_HS | High Score | Mataas na iskor |

When the game runs, it will look at what language the target device is running. If it is Filipino, it will take all of the text from the Filipino column. If it is any other language it will show English.

Timing of localisation will be dependent to an extent on how this is being done. If a commercial localisation company is being used we recommend waiting until after Beta before getting the text localised. This should mean that it can be done in two passes (main and pickup) which should minimise costs.

# 11.0 Test Plan

At the time of each key playable deliverables (technical prototype, alpha, beta and gold) a Test Plan will be created to ensure that testers know the main features that are to be checked.

The test plan will be developed by Charlotte Hemingway as it will also include measurements in knowledge and attitude relating to the game’s learning objectives.

Game builds used for test purposes will be distributed using either TestFlight or, preferably for ease/speed, the Google Play Beta System.

# Appendix 1: Key Deliverable Dates

**Activities**

| Activity No. | Activity | Responsibility | Start Date  (Project week) | End Date  (Project week) |
| --- | --- | --- | --- | --- |
| 1 | Decide and document the most appropriate specification and game design to support the scope. | EM Studios:  Bobby Farmer | (1) 01 Oct 16 | (13) 31 Dec 16 |
| 2 | Develop a Technical Prototype game | Bobby Farmer | (14) 01 Jan 17 | (25) 28 Feb 17 |
| 3 | Develop an Alpha build in line with the game design document and specification | Bobby Farmer | (26) 01 March 17 | (34) 30 April 17 |
| 4 | Develop a Beta build in line with suggested changes | Bobby Farmer | (35) 01 May 17 | (43) 30 June 17 |
| 5 | Develop Gold Master build ready for pilot delivery | Bobby Farmer | (44) 01 July 17 | (56) 30 Sept 17 |
| 6 | Technical support | Bobby Farmer | (57) 01 Oct 17 | (69) 31 Dec 17 |
